# Ruthenacycles and Iridacycles as Transfer Hydrogenation Catalysts

**DOI:** 10.3390/molecules26134076

**Published:** 2021-07-03

**Authors:** Vincent Ritleng, Johannes G. de Vries

**Affiliations:** 1 Ecole européenne de Chimie, Polymères et Matériaux, Université de Strasbourg, CNRS, LIMA, UMR 7042, 25 rue Becquerel, 67087 Strasbourg, France; 2 Leibniz Institut für Katalyse, e. V. Albert-Einstein Strasse 29a, 18059 Rostock, Germany

**Keywords:** metallacycle, ruthenium, iridium, transfer hydrogenation, oxidation, ketone

## Abstract

In this review, we describe the synthesis and use in hydrogen transfer reactions of ruthenacycles and iridacycles. The review limits itself to metallacycles where a ligand is bound in bidentate fashion to either ruthenium or iridium via a carbon–metal sigma bond, as well as a dative bond from a heteroatom or an *N*-heterocyclic carbene. Pincer complexes fall outside the scope. Described are applications in (asymmetric) transfer hydrogenation of aldehydes, ketones, and imines, as well as reductive aminations. Oxidation reactions, i.e., classical Oppenauer oxidation, which is the reverse of transfer hydrogenation, as well as dehydrogenations and oxidations with oxygen, are described. Racemizations of alcohols and secondary amines are also catalyzed by ruthenacycles and iridacycles.

## 1. Introduction

In this review, the use of cyclometalated complexes based on ruthenium and iridium in hydrogen transfer reactions is described. In the definition we use here, a cyclometalated complex has one anionic carbon–metal σ-bond and is additionally stabilized by a single intramolecular dative bond from the same ligand [1]. Thus, metal pincer complexes are outside the scope of this review.

## 2. Ruthenacycles as Transfer Hydrogenation Catalysts

The transfer hydrogenation (TH) of ketones is by far the most studied reaction with ruthenacycles, in particular with *CN*-ruthenacycles, as will become apparent in the following pages. Most of the published work deals with racemic reductions. Noteworthy, the most important contribution in asymmetric transfer hydrogenation comes from the work of Pfeffer and his collaborators. This review is dedicated to Michel Pfeffer for his pioneering work in this field.

### 2.1. Transfer Hydrogenation of Ketones

**Scheme 1:**
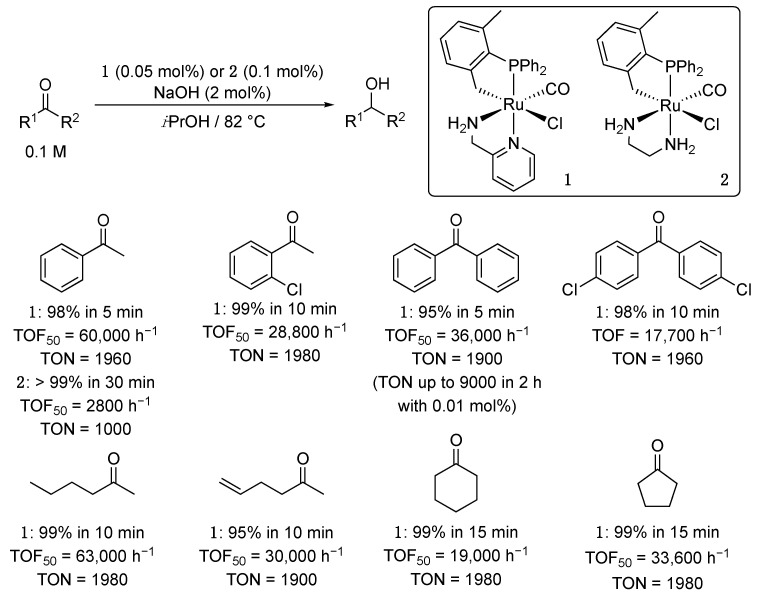
TH of ketones catalyzed by the *CP*-cycloruthenated complexes **1** and **2**.

The first examples of catalytic application of cyclometalated ruthenium complexes in ketone transfer hydrogenation appeared in 2004 in two successive contributions from the group of Baratta [2,3]. These reports followed a publication from van Koten in 2000 [4] that for the first time described interesting catalytic properties of a ruthenium *NCN*–pincer complex for TH. In these reports, Baratta et al. described the synthesis of a novel class of cycloruthenated complexes bearing the anionic (κ^2^-*C,P*)-[2-CH_2_-6-MeC_6_H_3_PPh_2_]^−^ ligand. These species resulted from the reaction of the 14-electron δ-agostic [RuCl_2_{(2,6-Me_2_C_6_H_3_PPh_2_)_2_] complex with formaldehyde in the presence of triethylamine via cyclometalation of an *ortho*-methyl group and aldehyde decarbonylation. Among the new cyclometalated complexes, the derivatives **1** and **2** bearing 2-(aminomethyl)pyridine (*ampy*) and ethylenediamine (*en*), respectively, were both found active for the TH of acetophenone (0.1 M) in isopropanol at reflux in the presence of NaOH (2 mol.%) as base and activator. When **2** (0.1 mol.%) was used, quantitative conversion to 1-phenylethanol was achieved after 30 min reaction, whereas, when **1** (0.05 mol.%) was used, 98% conversion was reached in only 5 min (Scheme 1). Thus, complex **1** (0.05 mol.%) was found to be highly active for the reduction of a number of aryl alkyl, diaryl, and dialkyl ketones achieving turnover frequencies at 50% conversion (TOF_50_) of up to 63,000 h^−1^ and turnover numbers as high as 9000 in 2 h when a loading of 0.01 mol.% was used (Scheme 1). These values were among the highest reported in the literature at the time [4,5,6,7,8].

Regarding the mechanism, it was postulated by the authors that **1** could react with the base to afford the corresponding amide complex that could subsequently react with isopropanol to yield the key ruthenium hydride amine species [9]. Alternatively, the latter could be generated through the alkoxide route [10]. Whatever the exact route, the high catalytic performance of **1** was tentatively ascribed to the combined presence of a bifunctional Ru–H/N–H motif [11,12] and of a stable Ru–C σ-bond that would prevent catalyst deactivation [2].

**Scheme 2:**
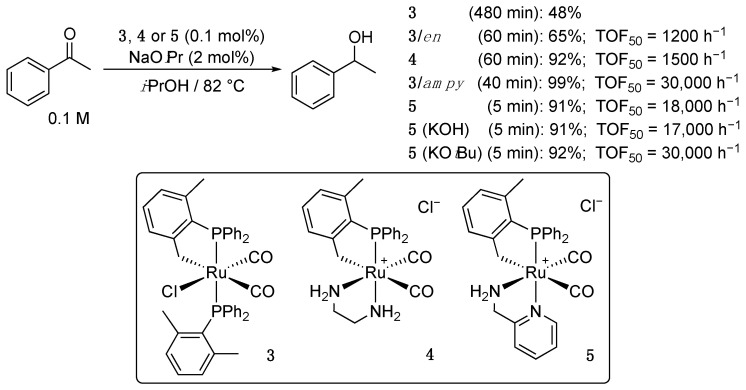
TH of acetophenone catalyzed by the dicarbonyl *CP*-cycloruthenated complexes **3**–**5**.

Baratta et al. recently extended the library of ruthenacyclic complexes bearing the anionic (κ^2^-*C*,*P*)-[2-CH_2_-6-MeC_6_H_3_PPh_2_]^−^ ligand by synthesizing the series of dicarbonyl derivatives **3**–**5** depicted in Scheme 2 [13]. The amine free complex **3** (0.1 mol.%) displayed poor activity in the TH of acetophenone (0.1 M) in the presence of NaO*i*Pr (2 mol%) as a base in 2-propanol at reflux, affording only 48% conversion into 1-phenylethanol after 8 h reaction. In situ addition of the bidentate ligands, *en* or *ampy* (2 equiv.), to **3** dramatically increased the catalytic activity of the latter, resulting in a TOF_50_ between 1200 and 30,000 h^−1^, thus suggesting an accelerating N–H effect upon coordination to the metal center. This was confirmed by the activity observed with the isolated cationic dicarbonyl species **4** and **5**, which were about the same as those observed with the in situ generated **3**/*en* and **3**/*ampy* systems, respectively (Scheme 2). Similarly to what was observed with the neutral mono carbonyl derivatives **1** and **2** [2,3], the *ampy* derivative **5** displayed the highest activity with TOF_50_ values ranging between 17,000 h^−1^ and 30,000 h^−1^ depending on the nature of the alkali base (NaO*i*Pr, KOH, or KO*t*Bu).

As for the mechanism, control experiments run with **4** at 85 °C under reduced pressure (10^−2^ mmHg) cleanly led to the formation of the mono-carbonyl complex **2**. This result, together with the fact that both **1** and **2** are twice as active as **5** and **4**, respectively, suggested a possible thermal dissociation of one CO ligand as the initial step of the reaction mechanism. This thermal CO displacement in the presence of 2-propanol and an alkali base would lead to the catalytically active Ru monohydride species that would then reduce acetophenone with the help of “*a hydrogen bonding network promoted by the NH_2_ function*” [13].

**Scheme 3:**
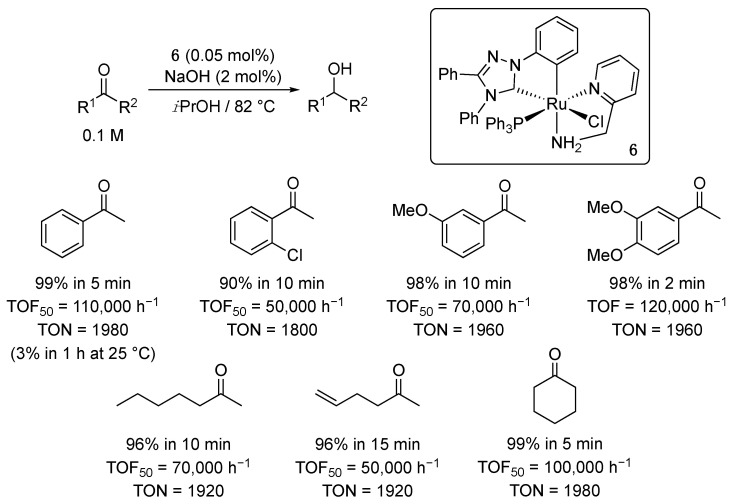
Highly efficient TH of ketones catalyzed by the *CC*-orthoruthenated complex **6**.

Following their work with the anionic (κ^2^-*C,P*)-[2-CH_2_-6-MeC_6_H_3_PPh_2_]^−^ ligand and the observation of the dramatic N–H accelerating effect upon coordination of *ampy* to the ruthenium center [2,3], Baratta and his collaborators reported the synthesis of complex **6** bearing an orthometalated phenyl-substituted *N*-heterocyclic carbene (NHC) ligand in combination with *ampy* [14]. In the presence of NaOH (2 mol.%) as a cocatalyst, **6** (0.05 mol.%) proved highly active for the TH of a small variety of aryl, alkyl, and dialkyl ketones from isopropanol at reflux, with TOF_50_ values ranging from 50,000 h^−1^ in the cases 2′-chloroacetophenone and 5-hexen-2-one to 120,000 h^−1^ in the case of 3′,4′-dimethoxyacetophenone (Scheme 3). The latter proved, thus, to be even more active than the *CP*-cyclometalated mono-carbonyl complex **1**, suggesting that the *CC*-orthometalated carbene with *ampy* is a particularly favorable combination for obtaining a highly active catalyst. It is noteworthy, however, that **6** showed almost no activity at room temperature. Furthermore, no activity was observed in the absence of a base, which suggested the intermediacy of a monohydride species that could be generated through the alkoxide/β-elimination route. The high catalytic performance of **6** would then, similarly to **1**, be due to the combined presence of an Ru–H/N–H motif and of a stable orthometalated ligand that would prevent catalyst deactivation. Regrettably, no mechanistic investigation was carried out to sustain these assumptions.

Surprisingly, other examples of *CC*-ruthenacycles comprising similar orthometalated aryl-substituted NHC ligands only appeared about a decade later.

**Figure 1:**
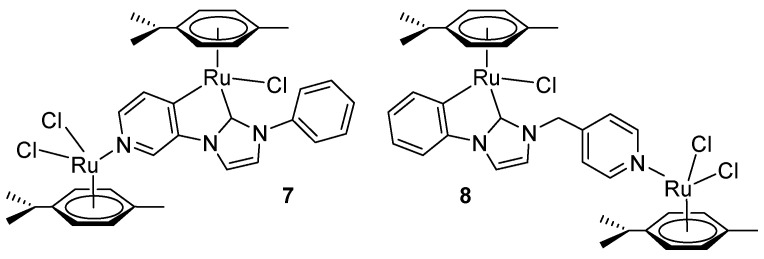
Bimetallic complexes **7** and **8** bearing a *CC*-cyclometalated NHC-pyridyl or NHC-phenyl ligand.

In a study aiming, among others, at establishing the influence of disparate electronic properties exerted by different cyclometalated groups on the catalytic activity of the resulting complexes, Choudhury et al. reported the synthesis, electronic characterization, and catalytic behavior of the bimetallic NHC-pyridyl and NHC-phenyl ruthenacycles **7** and **8**, depicted in Figure 1 [15]. The Ru^II^/Ru^III^ oxidation potential values measured for the cyclometalated ruthenium centers of **7** and **8** clearly established that the NHC-phenyl chelate is more electron-donating than the NHC-pyridyl chelate (0.812 V for **7** vs. 0.596 V for **8**). Both complexes (1 mol.%) proved to be moderately active for the TH of acetophenone (0.2 M) from isopropanol at 100 °C in the presence of a large amount of KOH (20 mol.%). Nevertheless, the electron-rich NHC-phenyl complex **8** was found to be a little more active than the relatively electron-poor NHC-pyridyl complex **7** with 92% conversion after 3.5 h reaction (TON = 92; TOF = 26.3 h^−1^) for the former and only 63% conversion after the same time for the latter (TON = 63; TOF = 18 h^−1^). A similar trend was observed with the corresponding IrCp* (Cp* = η^5^-C_5_Me_5_) complexes, which proved twice more active. Noteworthy, although complexes **7** and **8** possess two ruthenium centers in different environments, control experiments supported that the activity of the pyridine-coordinated metal center was significantly lower than that of the cyclometalated center.

**Scheme 4:**
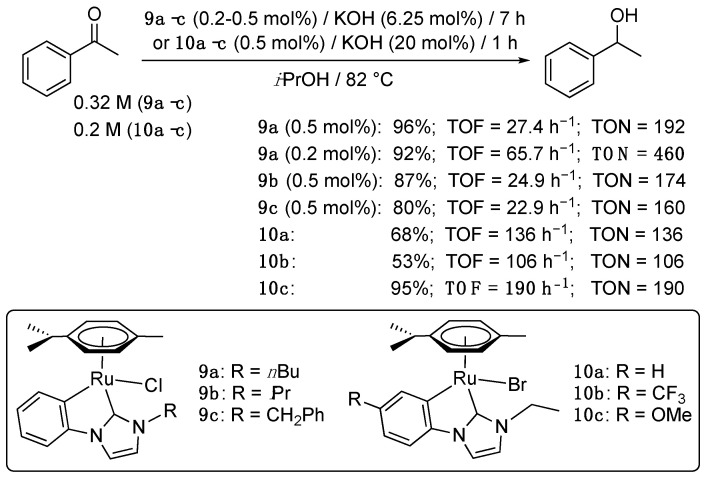
TH of acetophenone catalyzed by *CC*-cycloruthenated complexes **9a**–**c** and **10a**–**c**.

Monometallic complexes bearing closely related *CC*-cycloruthenated NHC-phenyl ligands were then successively described by the groups of Ramesh [16] and Rit [17], and studied for the TH of acetophenone from isopropanol at reflux. While Ramesh et al. studied the influence of the nature of the wingtip substituents in their series of three complexes **9a**–**c**, Rit et al. studied the influence of the nature of a substituent in *meta*-position to the C–Ru bond on the cyclometalated phenyl ring of complexes **10a**–**c** (Scheme 4). As the reaction conditions changed from one study to the other, notably regarding the amount of base (KOH) that varied from 6.25 mol.% with **9a**–**c** to 20 mol.% with **10a**–**c**, it is difficult compare the two series of complexes. Nevertheless, conclusions can be drawn within each series. Thus, among complexes **9**, the **9a** derivative, bearing an *n*-butyl substituent, proved to be more active than **9b** and **9c**, bearing bulkier isopropyl and benzyl wingtips, respectively [16]. Interestingly, the catalyst loading of **9a** could even be decreased to 0.2 mol.%, allowing a TON of 460, which is from far the highest value observed for the *CC*-cyclometalated complexes **7**–**10**, along with a much lower amount of KOH than in the cases of complexes **7**, **8**, and **10a**–**c** [15,16,17]. Among complexes **10**, the *m*-OMe derivative **10c** (0.5 mol.%) displayed the best activity, with 95% conversion to 1-phenylathanol after 1 h reaction (TOF = 190 h^−1^), and the *m*-CF_3_ derivative **10b** displayed the lowest, with only 53% conversion after 1 h (TOF = 106 h^−1^). This almost twofold difference in catalytic activity between **10c** and **10b** could be attributed to the presence of an electron-richer Ru(II) center in **10c**, as evidenced by the higher chemical shift of the carbene carbon in its ^13^C-NMR spectrum (δ(C_NHC_) = 185.4 (**10c**), 187.0 (**10a**), and 188.0 (**10b**) ppm), and by its lower Ru^II^/Ru^III^ redox potential (E_1/2_ = 72 mV (**10c**), 101 mV (**10a**), 224.5 mV (**10b**) vs. Fc) [17].

The reaction scopes of both **9a** (0.2 mol.%, KOH (6.25 mol.%)) and **10c** (0.5 mol.%, KOH (20 mol.%)) were studied under their respective optimized conditions. Noteworthy, both complexes were shown to reduce a large panel of ketones (16 substrates in each case), including aryl alkyl, diaryl, heteroaryl alkyl, and cyclic and acyclic dialkyl ketones, with good to excellent efficiency [16,17].

Mechanistic investigations in the case of **10a** resulted in evidence for the formation of a ruthenium hydride species upon reaction of **10a** with KOH in refluxing isopropanol, which suggested the possible intermediacy of such species in the catalytic cycle [17]. This assumption was further substantiated by the quantitative reduction of 4-chlorobenzaldehyde (see Section 2.4) to the corresponding alcohol when the latter was treated with the in situ generated ruthenium hydride. On this basis, the authors of this study proposed a classical monohydride mechanism, with formation of the ruthenium hydride intermediate by β-H-elimination from the corresponding ruthenium isopropoxide species.

**Figure 2:**
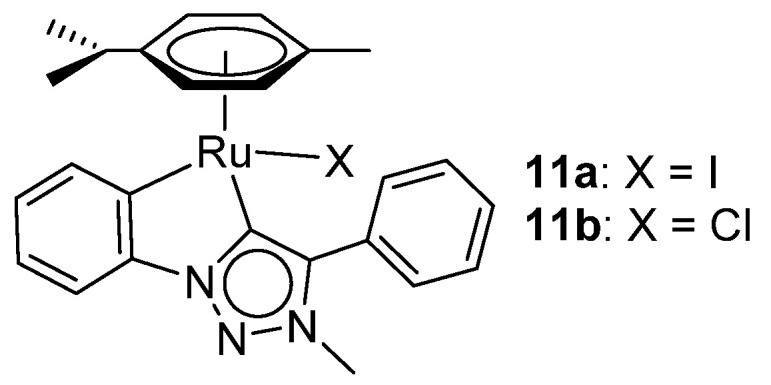
Poorly active and inactive *CC*-orthometalated triazolylidene Ru(II) complexes **11a** and **11b**.

In the same study, Rit et al. also reported the catalytic activity in various TH reactions of a related *CC*-ruthenacycle **11a** bearing an orthometalated mesoionic triazolylidene ligand (Figure 2). The latter (0.5 mol.%) displayed poor activity in ketone TH compared to complexes **9**–**10** with only 25% conversion of acetophenone (0.2 M) to 1-phenylethanol after 1 h reaction in the presence of KOH (20 mol.%) in refluxing isopropanol [17]. This poor activity, nevertheless, contrasted with the total absence of activity of its chlorinated analog **11b**, as reported by Albrecht et al. for the tentative TH of benzophenone (0.2 M) from isopropanol in the presence of 10 mol.% KOH under otherwise similar conditions [18].

**Scheme 5:**
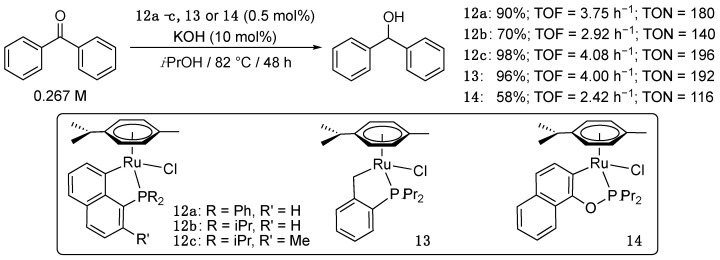
TH of benzophenone catalyzed by the *CP*-cyclometalated phosphine– or phosphinite–Ru(II) complexes **12a**–**c**, **13,** and **14**.

In 2017, Zhu et al. reported the synthesis of a series of half-sandwich five-membered *CP*-ruthenacyclic complexes **12a**–**c**, **13,** and **14** by intramolecular C(*sp*^2^)–H or C(*sp*^3^)–H activation of the corresponding phosphines or phosphinites with [Ru(η^6^-*p*-cymene)Cl_2_]_2_ in the presence of sodium acetate [19]. All complexes were fully characterized, and their reactivity was briefly studied. In particular, the authors evaluated the catalytic activity of complexes **12**–**14** for the TH of benzophenone (0.267 M) from isopropanol at reflux in the presence of KOH (10 mol.%) as a base. With the exception of the diisopropyl(1-naphtyl)phosphine complex **12b** and of the 1-naphtyl-diisopropylphosphinite derivative **14**, which proved less active, all complexes reduced benzophenone in 90% yield or more in 48 h with a catalyst loading of 0.5 mol.%, thus achieving TON of 180 or more, albeit with very low TOF values (Scheme 5). No mechanistic investigation was carried out.

**Scheme 6:**
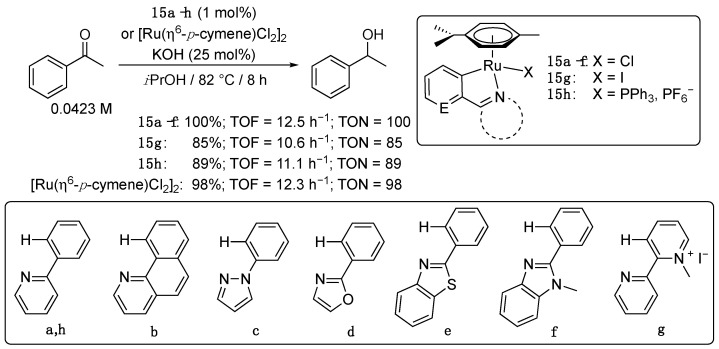
TH of acetophenone catalyzed by the *CN*-cycloruthenated complexes **15a**–**h**.

A series of eight half-sandwich *CN*-cycloruthenated complexes **15a**–**h** were similarly prepared by intramolecular C–H activation of the ligands displayed in Scheme 6 with [Ru(η^6^-*p*-cymene)Cl_2_]_2_ in the presence of potassium acetate, and investigated for their activity in the TH of aromatic ketones from isopropanol at reflux in the presence of KOH as a base [20]. Optimization studies established the necessity of using an amount of base as high as 25 mol.% to observe an appreciable reaction rate in the presence of 1 mol.% of **15a**–**h**. Under these conditions, complexes **15a**–**f** and [Ru(η^6^-*p*-cymene)Cl_2_]_2_ all achieved full conversion of acetophenone (0.0423 M) to 1-phenylethanol in 8 h. The slightly reduced activity observed with **15g** and **15h** was tentatively attributed to the low solubility of **15g** in isopropanol and to the substitution of the chloride ligand by triphenylphosphine in **15h**. Evaluation of the reaction scope of **15a**, **15e,** and **15f** revealed similar conversions for the 14 aromatic and heteroaromatic ketones investigated with the three complexes. Due to this similarity in activity and to the apparent absence of influence of the cyclometalated ligand, the authors suspected that the cycloruthenated complexes **15a**–**h** might be precatalysts undergoing a transformation to a common species acting as the catalyst. An array of control experiments, including stoichiometric NMR studies, kinetic monitoring, transmission electron microscopy (TEM), dynamic light scattering (DLS), and X-ray photoelectron spectroscopy (XPS), indeed allowed establishing the demetalation of the cyclometalated ligands under these basic conditions, as well as the formation of catalytically active Ru(0) nanoparticles [20].

**Scheme 7:**
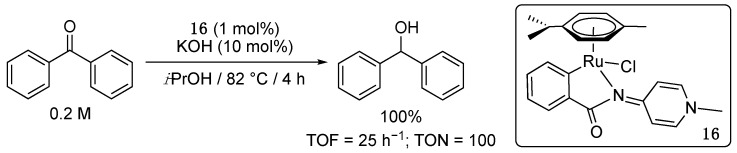
TH of benzophenone catalyzed by the *CN*-cyclometalated phenyl-pyridylideneamide complex **16**.

More recently, Albrecht et al. reported the synthesis and catalytic activity in ketone TH of the half-sandwich *CN*-ruthenacycle **16** bearing a hybrid chelating ligand comprising a pyridylideneamide nitrogen atom as one strong σ-donor site and an orthometalated phenyl ring as the second donor site [21]. The latter was compared to similar complexes bearing a pyridine, a pyridylidene, another pyridylideneamide, or a triazolylidene as the second donor site. A combination of NMR and cyclovoltammetric studies allowed establishing considerable electronic variation in this series of complexes with decreasing donor ability in the order phenyl > pyridylideneamide ~ triazolylidene ~ pyridylidene > pyridine. Interestingly, the cyclometalated phenyl complex **16** (1 mol.%) proved to be by far the most active precatalyst, achieving full reduction of benzophenone (0.2 M) in 4 h in the presence of 10 mol.% KOH in refluxing isopropanol (Scheme 7), whereas the others required 24 h to reach conversions ranging from 45% to 96% under the same conditions. Noteworthy, kinetic studies strongly suggested that all these complexes operate through a mononuclear reaction mechanism.

### 2.2. Base-Free Transfer Hydrogenation of Ketones Triggered by Cyclometalation

In addition to the above described classical examples of ketone TH with ruthenacycles in the presence of an alkali base, a couple of examples of base-free reactions, where the catalytic transformation was triggered by the cylometalation of a ligand, have also been reported.

**Scheme 8:**
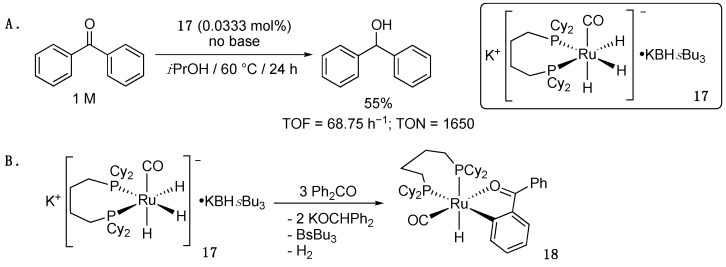
Base-free TH of benzophenone triggered by its orthometalation upon reaction with complex **17** (**A**) and orthometalation of benzophenone (**B**).

The first such example appeared as early as 2002 in a report from Fogg et al. [22]. In this report, mainly dedicated to H_2_ hydrogenation, the anionic trihydride complex K[Ru(Cy_2_P(CH_2_)_4_PCy_2_)(CO)H_3_]·KBH*s*Bu_3_ (**17**) (0.033 mol.%) was shown to achieve the TH of benzophenone to 1,2-diphenylethanol with 55% conversion after 24 h reaction in isopropanol at 60 °C in the absence of a base (Scheme 8A). Control experiments following hydrogenation reactions revealed the presence of a single species, identified by NMR, XRD, and elemental analyses as the ruthenacyclic monohydride complex **18** bearing an orthometalated benzophenone ligand. The same complex was also formed by reaction of **17** with a threefold excess of benzophenone (Scheme 8B). Both these results suggested that **18** was the actual TH catalyst.

**Scheme 9:**
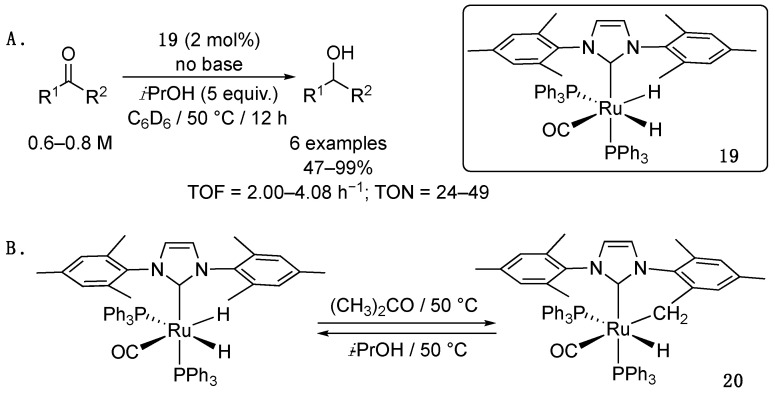
Base-free TH of ketones triggered by the orthometalation of the IMes ligand of complex **19** (**A**) and reversible orthometalation of the IMes ligand of complex **19** (**B**).

Another example of this type came from the group of Williams in 2005 with the base-free TH of several ketones catalyzed by the complex **19**, [Ru(IMes)(PPh_3_)_2_(CO)H_2_] (IMes = 1,3-dimesitylimidazol-2-ylidene), in benzene-*d*_6_ at 50 °C in the presence of 5 equiv. of isopropanol as the hydrogen donor (Scheme 9A) [23]. The latter was moderately efficient, achieving TON ranging only from 24 to 38 with aryl alkyl and diaryl ketones, as well as a TON of 49 with cyclohexanone in 12 h. Interestingly, however, a reversible C–H bond activation process was found to be at the origin of the catalytic transformation. Indeed, complex **19** was shown to undergo a facile dehydrogenation reaction in the presence of a ketone such as acetone or cyclohexanone at 50 °C to yield the cyclometalated complex **20** in which one C*sp*^3^–H bond of one mesityl group of the IMes ligand was activated. Furthermore, the starting complex **19** could be easily regenerated by the addition of 2-propanol to **20** at 50 °C (Scheme 9B).

**Scheme 10:**
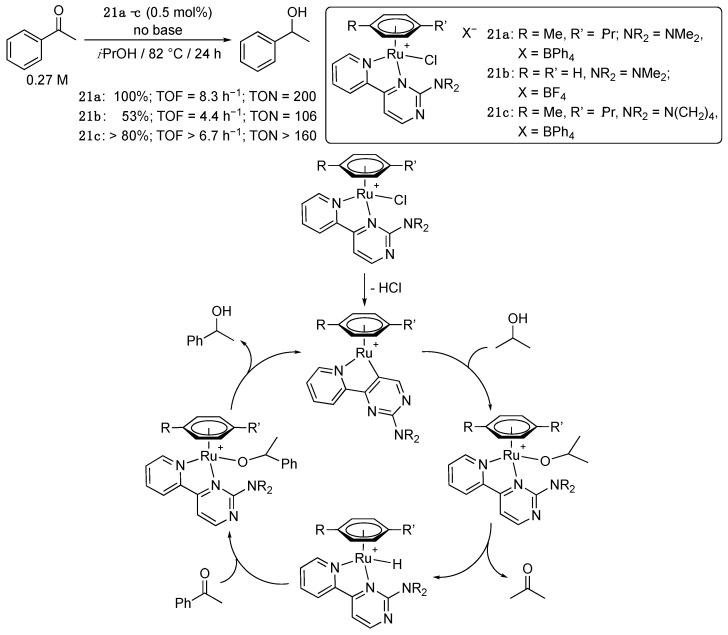
Base-free TH of acetophenone triggered by roll-over cyclometalation of the 2-(2-dialkylaminopyrimidin-4-yl)pyridine ligand of complexes **21a**–**c** and proposed mechanism.

A last example came from a series of two articles from Thiel et al. [24,25], in which (η^6^-arene)Ru(II) complexes **21a**–**c**, bearing a 2-(pyrimidin-4-yl)pyridine ligand substituted at the 2-position of the pyrimidinyl ring by a tertiary amine, were shown to catalyze the base-free TH of various ketones. As demonstrated by a combination of NMR spectroscopy, kinetic studies, collision-induced dissociation (CID) ESI-MS measurements, and DFT calculation, these base-free THs were triggered by the roll-over cyclometalation of the *N*,*N*′-chelating ligand leading to a 16-electron cyclometalated active species that would then follow a classical monohydride mechanism (Scheme 10). According to these data, the triggering roll-over cyclometalation was found especially favorable for 2-(pyrimidin-4-yl)pyridine ligands bearing a tertiary amine at the 2-position of the pyrimidine ring because the latter weakens the Ru–N′ bond by both steric and electric factors.

**Scheme 11:**
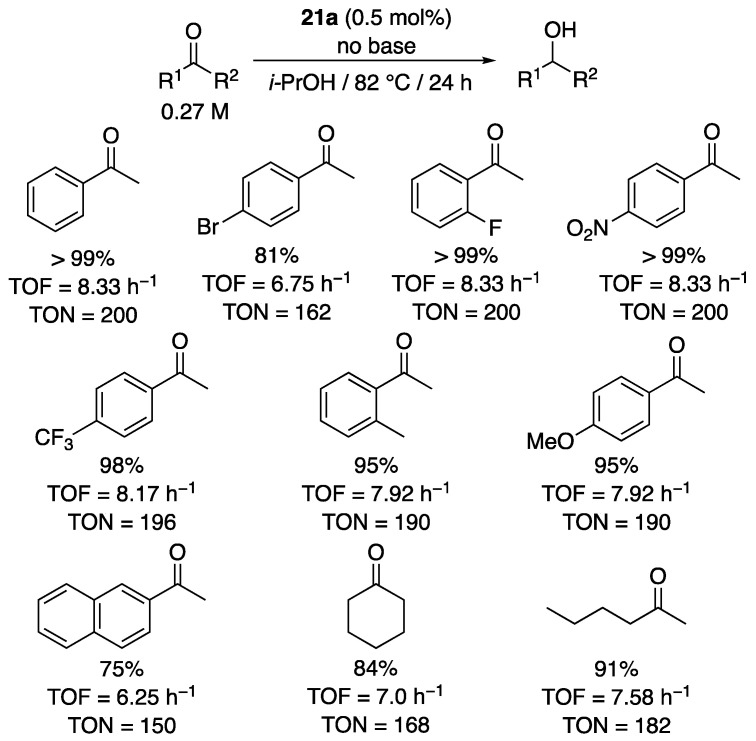
Base-free TH of ketones catalyzed by **21a**.

The most active 2-(pyrimidin-4-yl)pyridine complex **21a** was shown to efficiently catalyze the base-free reduction of 11 aromatic and aliphatic ketones (0.27 M) in isopropanol at reflux with a catalyst loading of 0.5 mol.% (Scheme 11). With the exception of methyl β-naphtyl ketone, all substrates were converted with yields over 80% in 24 h [25].

### 2.3. Asymmetric Transfer Hydrogenation of Ketones

**Scheme 12:**
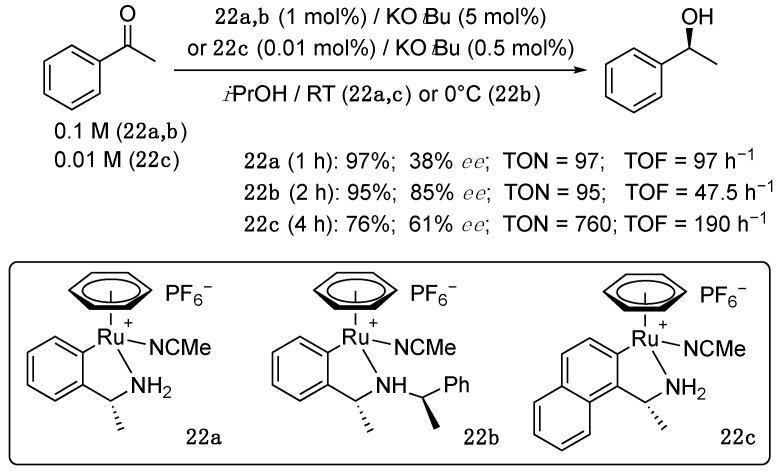
ATH of acetophenone with Pfeffer’s azaruthenacycles **22**.

The first example of application of ruthenacycles in asymmetric transfer hydrogenation (ATH) of ketones appeared in 2005 as a joint contribution of the groups of Michel Pfeffer in Strasbourg and Johannes G. de Vries at DSM [26]. This report, whose genesis started with a 2 month stay of Vincent Ritleng—then a PhD student of Michel Pfeffer—in DSM in 2000 [27], followed closely those from Baratta’s group [2,3], which described for the first time the use of ruthenacycles as efficient precatalysts for the same reaction in its racemic version (see Section 2.1). In this initial report, azaruthenacycles **22a**–**c** obtained by cyclometalation of enantiopure aromatic primary and secondary amines with [Ru(η^6^-C_6_H_6_)Cl_2_]_2_ were shown to be efficient catalysts for the asymmetric transfer hydrogenation of acetophenone (0.01–0.1 M) in isopropanol with TOF at the end of the reaction up to 190 h^−1^ at room temperature (**22c**, 0.1 mol.%) and enantiomeric excesses (*ee*) ranging from 38% (**22a**) to 85% (**22b**, 0 °C) (Scheme 12) [26].

**Scheme 13:**
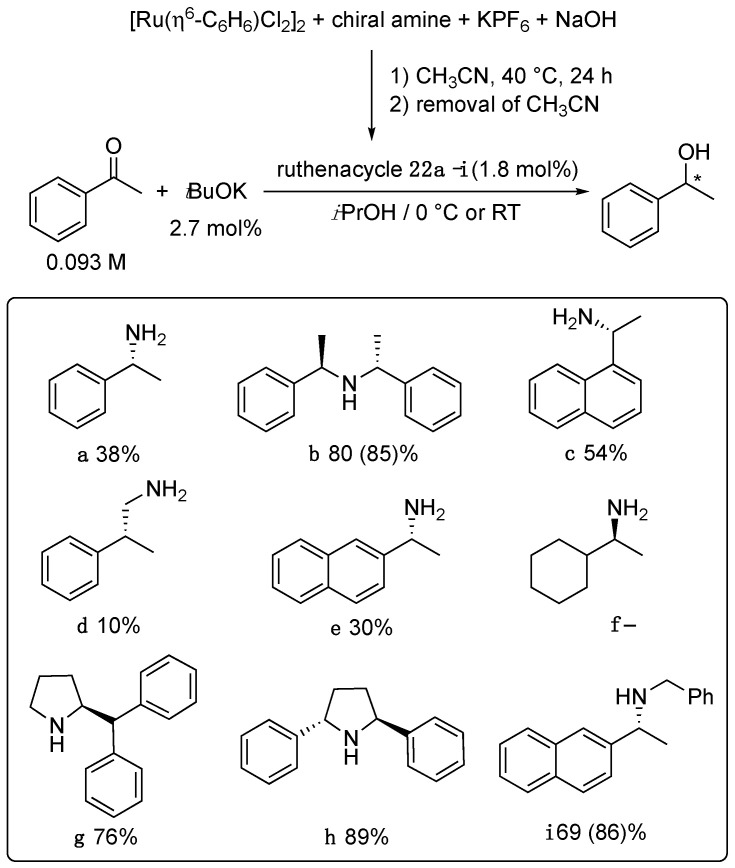
HTE screening of chiral amines in Pfeffer’s ATH of acetophenone (*ee* given below ligand structure; values between brackets obtained at 0 °C).

Interestingly, the synthesis of these ruthenacycles could be performed in situ, thus allowing the easy screening of a library of chiral primary and secondary amines through a high-throughput experiment (HTE) (Scheme 13) [26,28]. From this screening, it appeared that the majority of the ligands led to catalysts with interesting activity. In particular, the catalyst based on 1-naphtylethylamine **22c** turned out to be very fast, with TON and TOF values that could reach 10,000 and 30,000 h^−1^, respectively, at 80 °C with a loading of 0.01 mol.% [29]. Furthermore, the catalyst based on 2,5-diphenylpyrrolidine **h** induced the highest enantioselectivity (89%).

The lack of reactivity observed with the aliphatic amine **f** suggested the necessity to form a ruthenacycle to elicit transfer hydrogenation activity, the cyclometalation of **f** being obviously hampered by the lack of aromatic protons. Furthermore, the much reduced activity observed with the tertiary aromatic amine, *N*,*N*-dimethylbenzylamine [26], suggested a ligand cooperative mechanism involving the NH proton as in the Noyori transfer hydrogenation mechanism [11,12]. This assumption was later confirmed by a detailed mechanistic study [30] that allowed the identification of diastereomeric ruthenium-hydride intermediates and demonstrated the formation of a substrate–catalyst complex via hydrogen bonding between the O-atom of the substrate and the N–H unit of the catalyst and the subsequent hydride transfer to the substrate through a 6-membered transition state by an elegant combination of NMR studies and kinetic measurements.

A brief study of the reaction scope (nine ketones) with complexes **22b** and **22h** established that electron-deficient aromatic ketones such as 3,5-di-trifluoromethyl-acetophenone are hydrogenated very fast (TOF > 500 h^−1^) but with low enantioselectivity (29%) [31]. Branching in the α-position on the aliphatic side of the ketone led to much improved enantioselectivity, in particular with **22b** (89% to 98% *ee*). Tetralone also was reduced with excellent enantioselectivity (94%), but with catalyst **22h**. Lastly, both catalysts were able to reduce selectively, i.e., without reducing the C–C double bonds, 4-acetyl-styrene and 2-acetylfuran with very good enantioselectivity (86% to 87% *ee*) [32].

Since this series of papers from the Pfeffer and de Vries groups [26,27,29,30,31,32] were published, to our knowledge, only two more examples have appeared very recently which reported lower *ee*s: in 2018 from the group of Baratta, and in 2019 from that of Grabulosa.

**Scheme 14:**
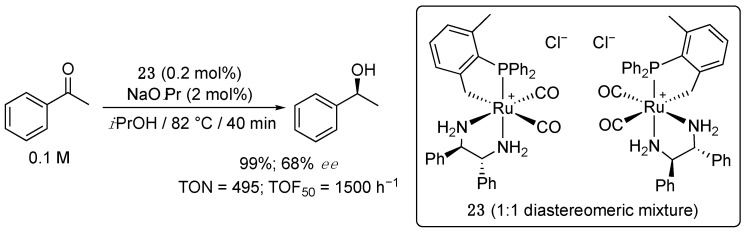
ATH of acetophenone catalyzed by the 1:1 diastereomeric mixture of the *CP*-cyclometalated dicarbonyl complex **23**.

As part of their work on *CP*-cyclometalated dicarbonyl complexes as racemic ketone TH catalysts (see Section 2.1, Scheme 2) [13], Baratta et al. also reported the synthesis of the chiral complex **23**, as a mixture of two diastereomers in a 1:1 ratio, by reaction of **3** in methanol at reflux with (*R*,*R*)-1,2-diphenylethylenediamine. In the presence of NaO*i*Pr (2 mol.%) in isopropanol at reflux, **23** (0.2 mol.%, as a diastereomeric mixture) quantitatively reduced acetophenone to (*S*)-1-phenylethanol in 40 min with a moderate *ee* of 68% (Scheme 14). When the reaction was carried out at lower temperature (60 °C), only 15% conversion was observed after 8 h, with no substantial increase in *ee*. By comparison with other ruthenium systems with (*R*,*R*)- or (*S,S*)-1,2-diphenylethylenediamine, which gave similar *ee*s in related hydrogenation reactions [33,34], the authors reasonably postulated that the enantioselectivity of this reduction is mainly controlled by the chiral ligand [13].

**Scheme 15:**
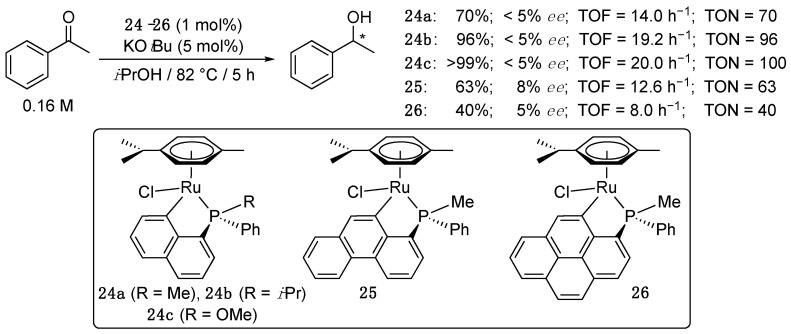
ATH of acetophenone catalyzed by *CP*-cycloruthenated complexes **24**–**26** with *P*-stereogenic monophosphines bearing a polycyclic aromatic substituent.

In 2019, Grabulosa and coworkers reported that the reactions of [Ru(η^6^-*p*-cymene)Cl_2_]_2_ with *P*-stereogenic 1-naphtyl-, 9-phenantryl-, or 1-pyrenyl-substituted phosphines in methanol in the presence of sodium acetate afforded the corresponding neutral cycloruthenated complexes **24**–**26** in low yields, and that the latter species could act as catalysts for the transfer hydrogenation of acetophenone after 15 min activation in isopropanol at 82 °C in the presence of KO*t*Bu [35]. The precatalyst **3c** bearing cyclometalated 1-naphtyl-substituted phosphinite showed the highest activity with full conversion already after 2 h reaction, but was completely unselective, similarly to the other 1-naphtyl-substituted monophosphine complexes **24a** and **24b** (Scheme 15). The bulkier 9-phenantryl- and 1-pyrenyl-substituted cycloruthenated phosphine complexes **25** and **26** showed lower activity with a very slightly enhanced enantioselectivity (5% to 8% *ee*). This quasi absence of enantioselectivity may be attributed to the existence of complexes **24**–**26** as diastereomeric mixtures. Thus, for instance, the non-cyclometalated dichloro-analogue of **24b**, gave a slightly higher *ee* of 13% for a conversion of 75% after 5 h under similar reaction conditions.

### 2.4. Transfer Hydrogenation of Aldehydes

**Scheme 16:**
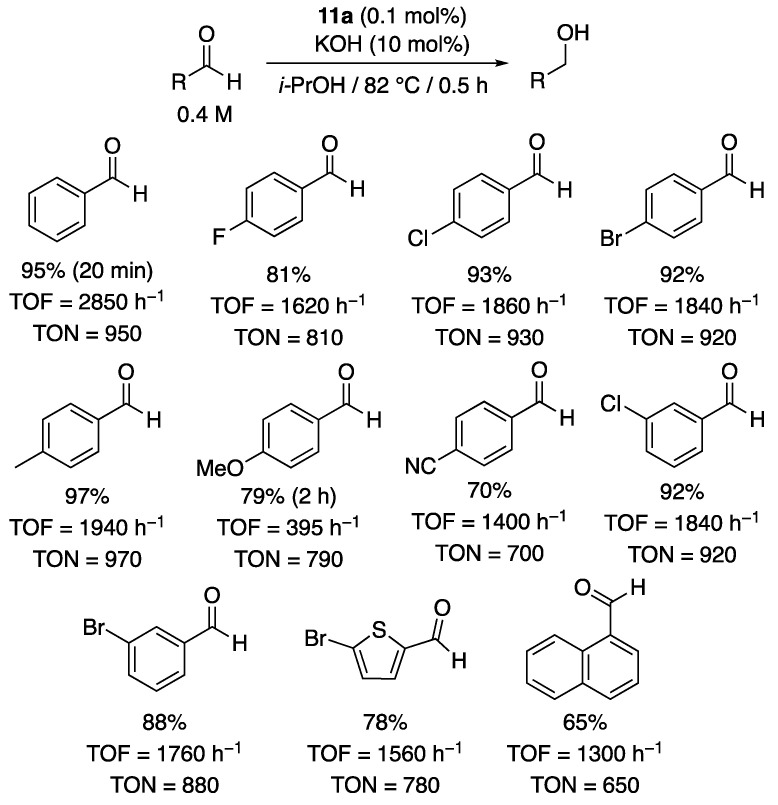
TH of aldehydes catalyzed by the cyclometalated triazolylidene-phenyl complex **11a**.

The transfer hydrogenation of aldehydes is rarely studied, and the catalytic efficiency is generally found to be very low with limited substrate scope along with the formation of aldol condensation or carboxylic acid byproducts [36]. We are, thus, aware of only one example of the use of a ruthenacycle as efficient catalyst for this transformation. In their extensive study of *CC*-cyclometalated NHC-phenyl- and triazolylidene-phenyl-Ru(II) complexes (see Section 2.1 and Section 2.5) [17], Rit et al. also evaluated the activity of ruthenacycles **10a**–**c** and **11a** for the TH of benzaldehyde. Whereas the NHC-phenyl species **10a**–**c** (0.1 mol.%, KOH (10 mol.%) in isopropanol at reflux) produced significant amounts of benzoic acid/benzoate as byproduct to benzyl alcohol, possibly through base-assisted Cannizaro reaction, the triazolylidene-phenyl complex **11a** produced benzyl alcohol in 95% yield under the same reaction conditions with no detectable side-product (Scheme 16). The reaction could be achieved within 20 min, giving a TOF of 2850 h^−1^.

The 3- and 4-substituted benzaldehydes containing both electron-withdrawing and electron-donating substituents were all effectively reduced to the corresponding alcohol (nine examples). Only 4-methoxybenzaldehyde required a longer reaction time (TOF = 395 h^−1^), probably due to the reduced electrophilicity of its aldehyde moiety. In addition, the heteroaromatic substrate, 5-bromo-2-thiophene, was also reduced efficiently, and the bulky 1-naphtylaldehyde was converted to the corresponding alcohol in 65% yield.

### 2.5. Transfer Hydrogenation of Aldimines

**Scheme 17:**
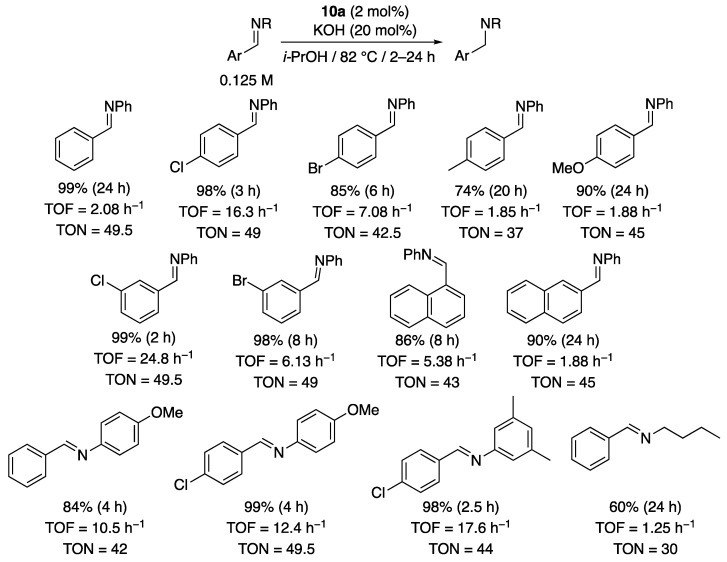
TH of aldehydes catalyzed by the cyclometalated triazolylidene-phenyl complex **10a**.

Despite the importance of amines for the synthesis of bioactive compounds, agrochemicals, fragrance, and industrially relevant polymers, ruthenium-catalyzed TH of aldimines has, comparatively to ketones, only been scarcely reported [23], and we are, in this case also, aware of only one example using ruthenacycles that comes from the work of Rit et al. [17]. Similarly to what was observed with ketones (see Section 2.1, Scheme 4), the electron-rich *p*-methoxy-substituted NHC-phenyl-Ru(II) complex **10c** (2 mol.%, KOH (20 mol.%)) performed better than the electron-poor *p*-trifluoromethyl-substituted derivative **10b** and the electron-neutral derivative **10a**, achieving full reduction of *N*-benzylideneaniline to *N*-benzylaniline (98%) in 10 h in isopropanol at reflux (vs. 99% in 24 h with **10a** and 73% in 24 h with **10b**). In accordance with its low activity in ketone reduction, the triazolylidene-phenyl derivative **11a** proved also ineffective in this transformation (17% conversion in 24 h). Substantial decomposition of the imine products to the corresponding anilines and aldehydes/alcohols was, however, observed in some cases with **10c**, and the substrate scope was studied with **10a** (Scheme 17). Electron-withdrawing substituents at the 3- or 4-position of the *C*-phenyl ring of aldimines substantially enhanced the reaction rate with TOF’s ranging from 6.1 to 24.8 h^−1^ whereas electron-donating substituents at the 4-position of *C*-phenyl ring slowed down the reaction with TOF values around 1.8 h^−1^ vs. 2.08 h^−1^ for *N*-benzylideneaniline. Variations of the substituents on the *N*-phenyl ring had less effects on the reaction rate. 1-Naphtyl-based aldimine (TOF = 8.38 h^−1^) was reduced faster than 2-naphtyl-based aldimine (TOF = 1.88 h^−1^). Lastly, in contrast to the *N*-aromatic aldimines that were screened, the *N*-alkyl aldimine, *N*-benzylidene butylamine, proved a relatively poor substrate for this catalytic system with only 60% conversion to the corresponding amine after 24 h reaction.

### 2.6. Asymmetric Transfer Hydrogenation of Imines

**Scheme 18:**
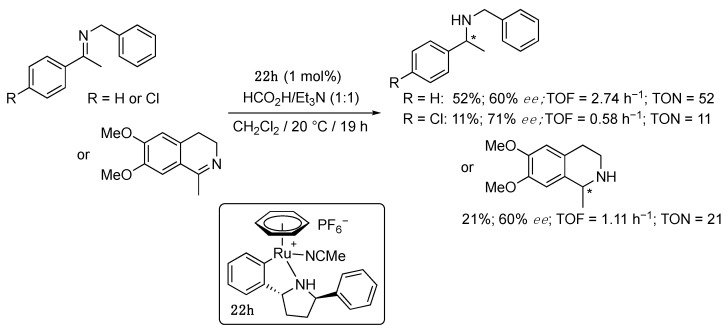
ATH of imines with Pfeffer’s azaruthenacycle **22h**.

Among the methods available to generate optically active amines, asymmetric hydrogen transfer on prochiral imines is of high economic and fundamental importance. Nevertheless, the only known example to date with half-sandwich ruthenacycles comes from the groups of de Vries and Pfeffer with the *CN*-metalacyclic complex **22h**. In their brief study, they showed that **22h** was able to reduce the three ketimines displayed in Scheme 18 with low to moderate yields and reasonable enantioselectivity in dichloromethane at 20 °C in the presence of a dry 1:1 mixture of formic acid and triethylamine as the hydrogen source [32]. Of note, among the tested complexes **22**, **22h** was the only one to give *ee*s superior to 40%. In particular, the catalysts derived from the primary amines led to amines with *ee*s below 20%. These results were in line with the mechanistic studies performed with acetophenone that showed that ruthenacycles based on chiral primary amines were unable to induce high *ee*s because of the formation of diastereomeric Ru hydride intermediates that display competitive rates but opposite enantioselectivities [30].

### 2.7. Transfer Hydrogenation of Alkynes and Alkenes

Examples of ruthenium-catalyzed TH of alkynes and/or alkenes are exceedingly rare, and we are aware of only two examples with ruthenacycles, one that deals with the partial hydrogenation of alkynes to alkenes [37] and another one that deals with the TH of alkenes to alkanes [38].

**Scheme 19:**
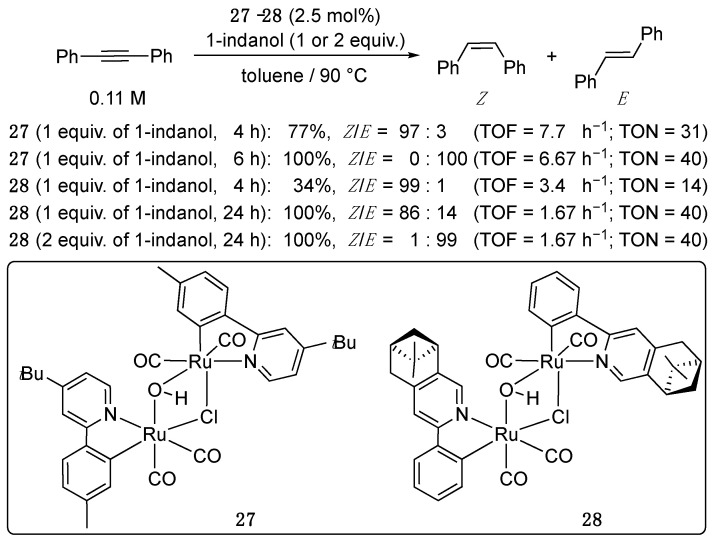
Base-free TH of diphenylacetylene to *Z*- and/or *E*-stilbene catalyzed by μ-chlorido,μ-hydroxy-bridged dicarbonyl ruthenacycles **27** and **28**.

In a very complete and elegant study, Djukic and Pfeffer reported that the μ-chlorido, μ-hydroxy-bridged dicarbonyl ruthenacycles **27** and **28** (2.5 mol.%) behaved as efficient precatalysts for the TH of diphenylacetylene to stilbene from 1-indanol in toluene at 90 °C in the absence of a base (Scheme 19) [37]. Complexes **27** and **28** were obtained by the highly stereoselective reaction of their μ-dichlorido bridged congeners with water in the presence of Na_2_CO_3_. Noteworthy, the μ-dichlorido-bridged precursors could also achieve the partial TH of diphenylacetylene and a couple of other diarylalkynes in the presence of water and of a soft inorganic base, ingredients necessary for the production of the μ-chlorido, μ-hydroxy-bridged complexes **27** and **28**.

Owing to its higher solubility in toluene, complex **27** showed a higher activity than **28**, achieving full partial hydrogenation to *Z*-stilbene and subsequent isomerization to *E*-stilbene in 6 h. The *Z*–*E* isomerization of alkenes was favored by the use of an excess amount of 1-indanol as shown by the formation of 86:14 and 1:99 *Z*/*E* mixtures after 24 h reaction with **28** in the presence of 1 and 2 equiv. of 1-indanol, respectively (Scheme 19).

**Scheme 20:**
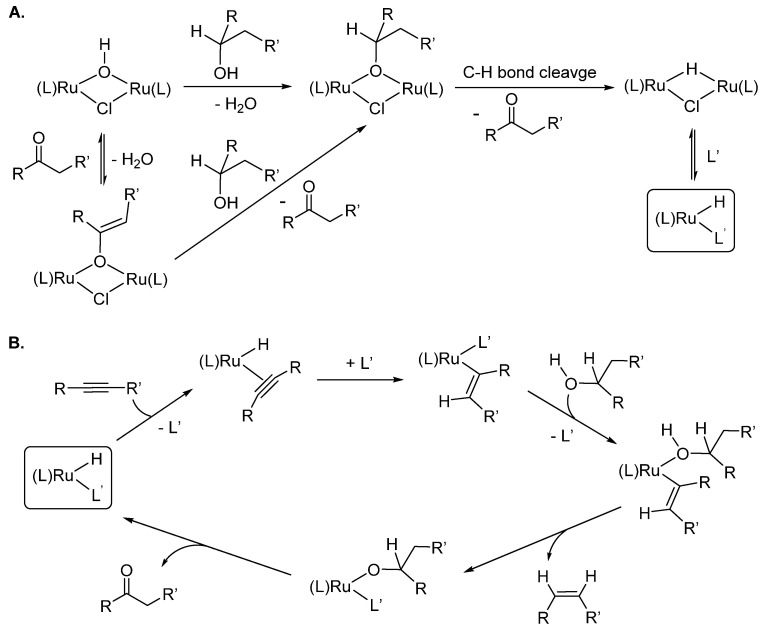
Generation of the catalytically active ruthenium hydride species from the μ-chlorido,μ-hydroxy-bridged ruthenacycles **27** and **28** (**A**) and partial TH of alkynes (**B**).

Regarding the mechanism, complexes **27** and **28** were shown, upon reaction with secondary alcohols, to produce ruthenium hydride species that are believed to be the active species. In addition, the determination of a *k*_H_/*k*_D_ isotopic effect of 7.0 ± 0.2 with 1-*d*-1-indanol allowed the authors to establish that the partial hydrogenation of diphenylacetylene relies on the hydrogen transfer from 1-indanol via C–H bond cleavage of a putative alkoxy ligand coordinated to the Ru(II) center in a rate-determining step. Furthermore, a control experiment performed with 1-indanone and D_2_O in the presence of **28**, which resulted in 40% deuterium incorporation at the methylene C in the α-position to the carbonyl, suggested that 1-indanone is not innocent and that it may contribute to the overall catalytic process by facilitating the formation of the ruthenium hydride from the μ-chlorido,μ-hydroxy-bridged precatalyst through the formation of ruthenium enolates. On the basis of these results, the authors proposed the mechanisms depicted in Scheme 20 for the generation of the catalytically active ruthenium hydride species (Scheme 20A) and the partial TH of alkynes (Scheme 20B).

**Scheme 21:**
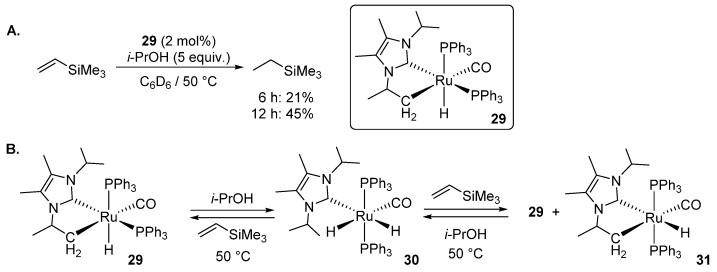
Base-free TH of trimethylvinylsilane catalyzed by the hydrido-ruthenacycle **29** (**A**) and reversible C–H bond activation of the carbene ligand (**B**).

The only example of TH of alkenes that we are aware of comes from the group of Williams, who reported in 2007 that the C–H activated carbene complex **29** could achieve the base-free TH of trimethylvinylsilane using isopropanol as reductant in benzene-*d*_6_ at 50 °C with moderate efficiency (Scheme 21A) [38]. Similarly to what was observed with complexes **19**/**20** [23], the key to the hydrogen transfer chemistry of **29** appeared to be the reversible C–H bond activation of the coordinated carbene ligand. Indeed, reactions of the cyclometalated complex **29** with isopropanol produced the dihydride complex **30** bearing the non-metalated carbene, and, when a sample of **30** was reacted with excess trimethylvinylsilane at 50 °C, **29** was regenerated along with an isomer **31** bearing the triphenylphosphine ligands in *trans*. The latter could similarly be converted back to **30** by reaction with isopropanol (Scheme 21B). This, together with control experiments of phosphine ligand exchange, suggested that these species are able to react by facile phosphine dissociation, which, in addition to the reversible C–H activation process, is likely to be relevant to the observed catalytic activity.

### 2.8. Oppenauer Alcohol Oxidation

The reverse reaction of ketone TH, i.e., alcohol oxidation to the ketone using a sacrificial ketone (usually acetone) as a hydrogen acceptor, has been much less studied, probably because the extent of alcohol oxidation is limited by the attainment of an equilibrium point based on the relative oxidation potentials of the ketone product and of the sacrificial ketone [39]. Thus, only a couple of examples have been reported with ruthenacycles.

**Scheme 22:**
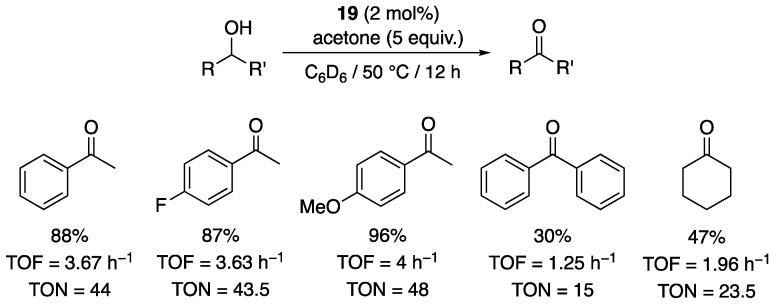
Alcohol oxidation by base-free TH with acetone catalyzed by **19**.

A first example appeared in 2005 with the carbene complex **19**, which, in addition to the base-free TH of ketones (see Section 2.2, Scheme 9A), was shown to catalyze the oxidation of a small set of secondary alcohols into ketones using acetone as the hydrogen acceptor (Scheme 22) [23]. Owing to the reversible C–H bond activation process leading to complex **20** in the presence of a ketone and leading back to **19** in the presence of an alcohol (Scheme 9B), the reaction occurred in the absence of a base.

**Scheme 23:**
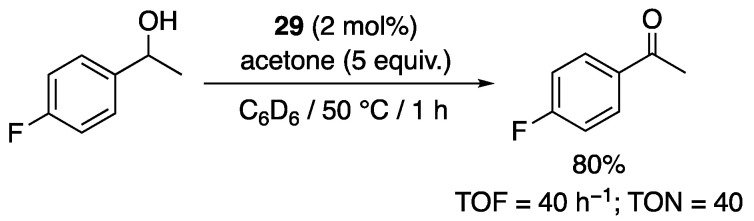
Oxidation of 4-fluoro-α-methylbenzyl alcohol by base-free TH with acetone catalyzed by the hydrido-ruthenacycle **29**.

Another base-free alcohol oxidation by TH was reported 2 years later by the same group with the hydrido-ruthenacycle **29** (2 mol.%) that was shown to yield 80% oxidation of 4-fluoro-α-methylbenzyl alcohol to the corresponding ketone in only 1 h at 50 °C in benzene-*d*_6_ in the presence of 5 equiv. of acetone, thus achieving a TOF of 40 h^−1^ (Scheme 23) [38].

**Scheme 24:**
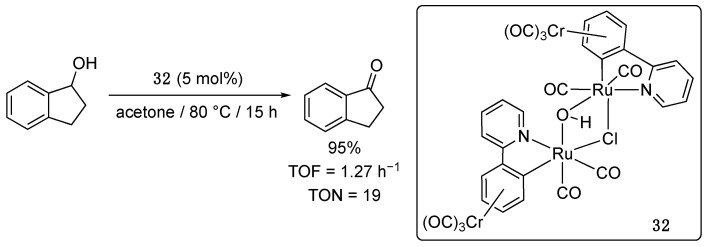
Oxidation of indanol by base-free TH with acetone catalyzed by the μ-chlorido,μ-hydroxy-bridged ruthenacycle **32**.

The μ-chlorido,μ-hydroxy-bridged homolog **32** of the alkyne TH catalysts **27** and **28** was shown in an earlier communication by Djukic and Pfeffer to catalyze the related Oppenauer oxidation of indanol in acetone at 80 °C with a catalyst loading of 5 mol.% in the absence of a base (Scheme 24) [40]. In agreement with their work with the ruthenacycles **27** and **28** (see Scheme 19 and Scheme 20) [37], the authors suggested that the process could rely on the formation of a ruthenium-hydride species.

### 2.9. Alcohol Racemization and Dynamic Kinetic Resolution

Dynamic kinetic resolution (DKR), in which rapid metal-catalyzed racemization of the undesired enantiomer is coupled with an enzymatic kinetic resolution, is an attractive methodology to obtain valuable enantiomerically pure amines and alcohols in 100% yield [41,42,43]. To this end, a small number of the ruthenacycles initially developed for ketone TH (or closely related analogues) have been studied in alcohol racemization.

**Scheme 25:**
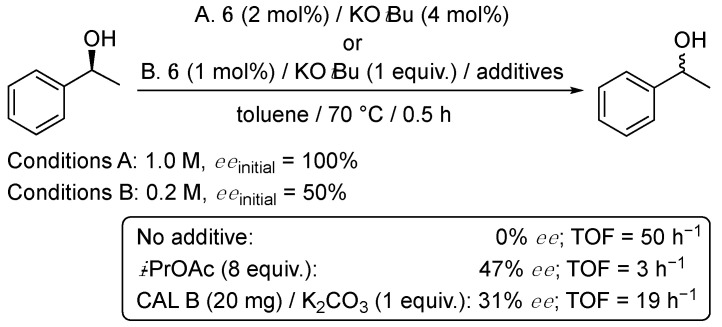
Racemization of (*S*)-1-phenylethanol catalyzed by the *CC*-ruthenacycle **6** under various conditions.

The exceptionally active ketone TH catalyst **6** (2 mol.%), initially reported by Baratta et al. (see Scheme 3) [14], was shown by Arends and collaborators to achieve the full racemization of (*S*)-1-phenylethanol in 30 min in toluene at 70 °C in the presence of KO*t*Bu (4 mol.%) as activator (Scheme 25) [44]. However, under DKR conditions, i.e., when the lipase CAL B or the acyl donor *i*-PrOAc was present, the active species resulting from **6** was greatly deactivated, and enantiomeric excesses of 31% and 47% were attained after 30 min reaction (Scheme 25).

**Scheme 26:**
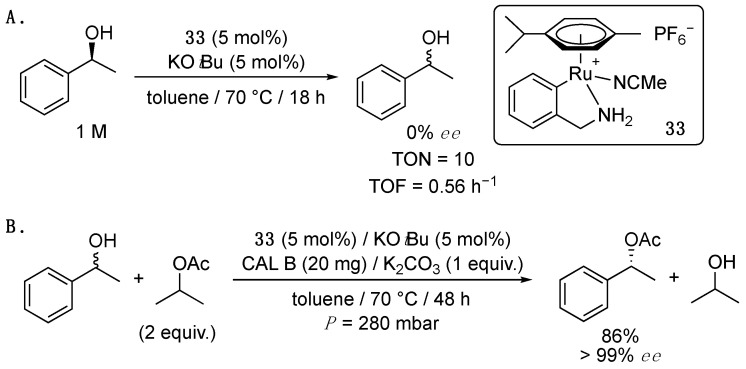
Racemization of (*S*)-1-phenylethanol catalyzed by the *CN*-ruthenacycle **33** (**A**) and DKR of 1-phenylethanol catalyzed by **33** and CAL B (**B**).

In the same report, Arends et al. described the synthesis of the achiral analogue **33** of the azaruthenacycle **22a** (see Scheme 12) [26] and its use for the racemization of (*S*)-1-phenylethanol [44]. The cycloruthenated benzylamine complex **33** (5 mol.%) displayed a reasonable activity compared to **6**, achieving full racemization of (*S*)-1-phenylethanol in 18 h at 70 °C in the presence of 5 mol.% KO*t*Bu (Scheme 26A). Furthermore, **33** proved stable under DKR conditions, and *rac*-1-phenylethanol was converted at 96% in 86% enantiopure (*R*)-1-phenylethyl acetate after 48 h reaction by using a catalytic combo of **33** and CAL B, with the only byproduct being acetophenone (Scheme 26B).

**Scheme 27:**
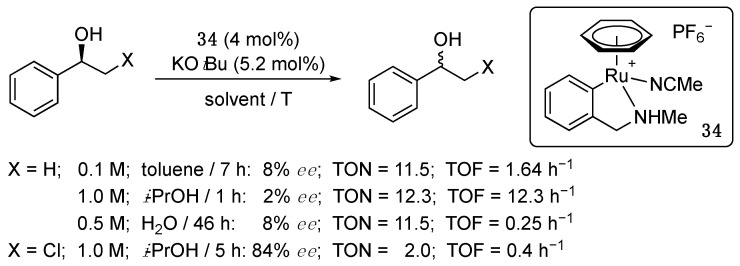
Racemization of (*S*)-1-phenylethanol catalyzed by the *CN*-ruthenacycle **34** under various conditions.

The closely related derivative **34** (4 mol.%) was similarly shown by Pfeffer and de Vries to catalyze the racemization of (*S*)-1-phenylethanol in the presence of KO*t*Bu (5.2 mol.%) [31,45]. Interestingly, the reaction could be carried out in various solvents at RT including water (Scheme 27). The *CN*-ruthenacycle **34** proved, however, inefficient for the racemization of (*R*)-2-chloro-1-phenylethanol, which prevented its use for the chemo-enzymatic DKR of β-haloalcohols to afford enantiopure epoxides with the help of a haloalcohol dehalogenase [46].

**Scheme 28:**
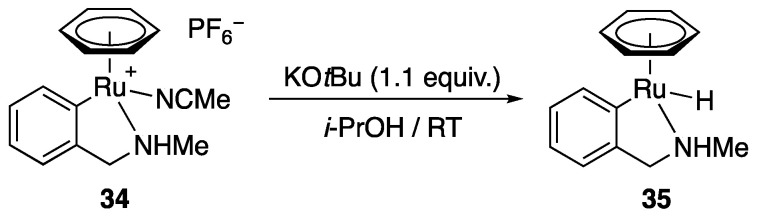
Formation of the racemization active species **35**.

A control experiment demonstrated that the deactivation of **34** in the racemization of (*R*)-2-chloro-1-phenylethanol was due to the formation of small amounts of 2-chloroacetophenone, which react almost instantaneously with the ruthenium hydride active species **35**—generated by action of KO*t*Bu on **34** (Scheme 28)—to yield a novel cycloruthenated species, whose exact nature could not be determined [45].

## 3. Iridacycles as Transfer Hydrogenation Catalysts

A mini-review about the use of iridacycle Cp*-complexes as catalysts for the production of fine chemicals was published in 2018 [47]. Wang and Xiao published an account of their own work on the use of iridacycles for (transfer) hydrogenation and dehydrogenation reactions [48].

### 3.1. Transfer Hydrogenation of Ketones

**Scheme 29:**
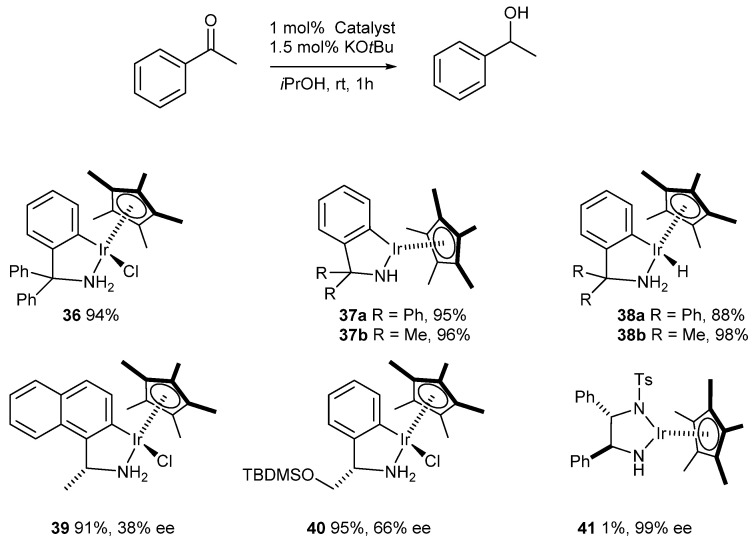
Performance of iridacycles **36**–**41** as catalysts in the TH of acetophenone.

Ikariya and coworkers synthesized iridacycles **36**, **39,** and **40** via reaction between [Cp*IrCl_2_]_2_ and the appropriate benzylamines in CH_2_Cl_2_ at room temperature in the presence of NaOAc. Treatment of these complexes with KO*t*Bu led to formation of the 16 electron amide complexes, such as **37**. Reaction of these with isopropanol resulted in the hydride complexes such as **38**. These complexes were tested in the TH of acetophenone (Scheme 29, percentages indicate yield of 1-phenylethanol) [49]. Reactions using the chloride complexes were performed in the presence of 1.5 equiv. of KO*t*Bu. For comparison, the same reaction was performed using the classical Noyori–Ikariya iridium TH catalyst **41** in the presence of KO*t*Bu. It turned out that the reactions using the iridacycles were much faster than the one catalyzed by **41**, although the enantioselectivity of the product 1-phenylethanol was substantially lower upon use of the chiral analogues **39** and **40**.

**Scheme 30:**
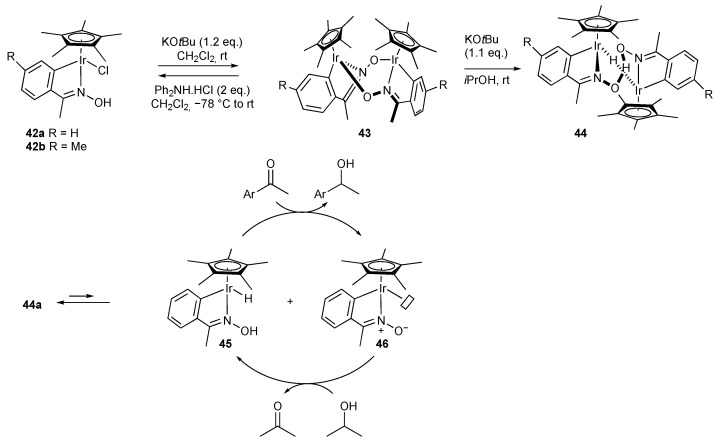
Oxime-based iridacycles as catalysts for the TH of acetophenones.

In a similar vein, Kuwata, Ikariya, and coworkers prepared iridacycles **42** by reacting acetophenone oximes with [Cp*IrCl_2_]_2_ and NaOAc in CH_2_Cl_2_ at room temperature. Treatment with base in CH_2_Cl_2_ led to the dimeric complexes **43** (Scheme 30). Upon treatment with base in isopropanol, these dimers formed the monohydride complexes **44** that were moderately active as TH catalyst [50]. The authors assumed that the dimer **44a** can dissociate in a monohydride **45** and complex **46**. Complex **46** can be reduced by isopropanol into **45**. These catalysts were used for the TH of a small set of substituted acetophenones. However, for decent conversions, 5 mol.% of catalyst was needed at a temperature of 50 °C and a duration of 15 h. These results are in stark contrast to the results obtained with the amine-based iridacycles of Scheme 29, which were orders of magnitude faster.

**Scheme 31:**
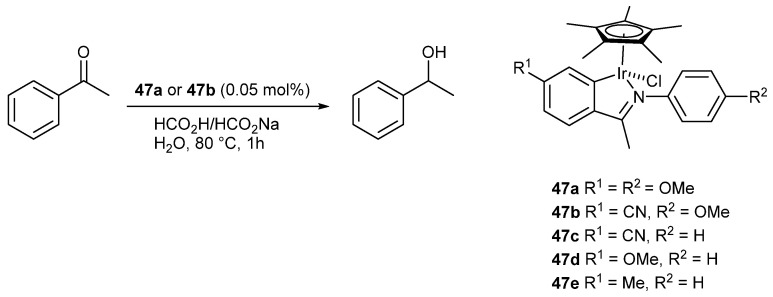

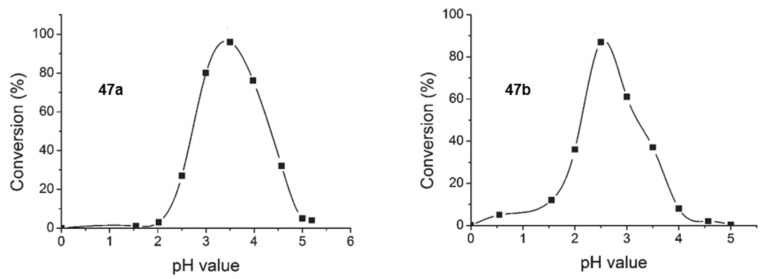
pH dependency of TH with imine-based iridacycles **47a** and **47b** (graphs reproduced from [51] with permission from the Royal Society of Chemistry).

Xue, Xiao, and coworkers examined the use of iridacycles **47a**–**e** ligated by acetophenone-*N*-aryl-imines as TH catalyst for aldehydes and ketones in water, using formate salts as reductant [51]. The authors found a strong dependence of the rate of the reaction on the pH of the aqueous solution (Scheme 31). The optimum pH was in the acidic region. This was explained by the authors to be necessary for the activation of the ketone as this type of catalyst does not have an NH available that can form a hydrogen bond with the ketone oxygen atom.

All catalysts were screened in the TH of acetophenone. Highest yields of 1-phenylethanol were obtained with the cyanide containing catalysts. Thus, catalyst **47c** was used for the reduction of a range of substituted acetophenones and aliphatic ketones, such as cyclohexanone and 2-octanone, as well as a range of substituted benzaldehydes, to alcohols with yields ranging from 91–100%. These reactions were performed at an S/C ratio of 2000 at pH 2.5 and 80 °C and took either 4 or 12 h.

**Scheme 32:**
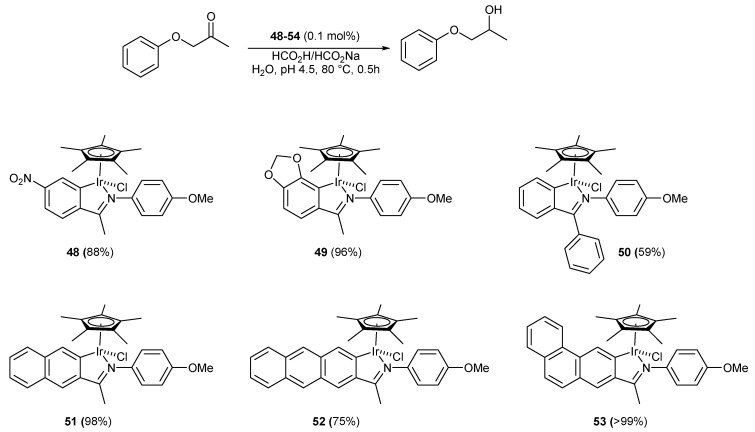
Comparison of iridacycle catalysts **48**–**53** in the transfer hydrogenation of 1-phenoxyacetone (yields of the alcohol in brackets).

The Xiao group synthesized another six iridacycles **48**–**53** and applied these in the TH of α-substituted ketones using formate salts as reductant in water [52]. Initially, the six catalysts were compared in the TH of 1-phenoxyacetone, by measuring the yield of the product alcohol after 0.5 h at 0.1 mol.% of catalyst and 80 °C (Scheme 32). Catalyst **53** was so active that it was also tested at lower catalyst/substrate ratios; at S/C = 10,000, it was still possible to reach 99% conversion after 2 h. This catalyst (**53**) was then used for the TH of a range of aryloxyacetophenones, perfluoroalkoxy-acetophenones, aryloxyacetones, and aliphatic 2-alkoxy-ketones using the conditions of Scheme 32, but at 0.01 mol.% of catalyst for 14 h. Excellent isolated yields were reported for all product alcohols (86–97%). Catalyst **51** (0.1 mol.%) was next used for the reduction of a range of acetophenones, α-substituted with hydroxy, chloro, dichloro, fluoro, trifluoro, nitrile, ester, *N*-morpholino, and dimethoxy, at 80 °C over 18 h. The product alcohols were obtained in yields ranging from 86–96%. The same catalyst (0.1 mol.%) was also used for the reduction of α- and β-ketoesters (80 °C, 14 h) to the hydroxyesters in yields from 91–96%. Catalyst **51** (0.1 mol.%) was not selective for the reduction of α,β-unsaturated ketones; however, it was capable of reducing the carbonyl group selectively in the reduction of α,β-unsaturated aldehydes (80 °C, 6 h). A number of cinnamaldehyde and aliphatic α,β-unsaturated aldehydes were reduced selectively to the allylic alcohols with yields from 78–92%.

**Scheme 33:**
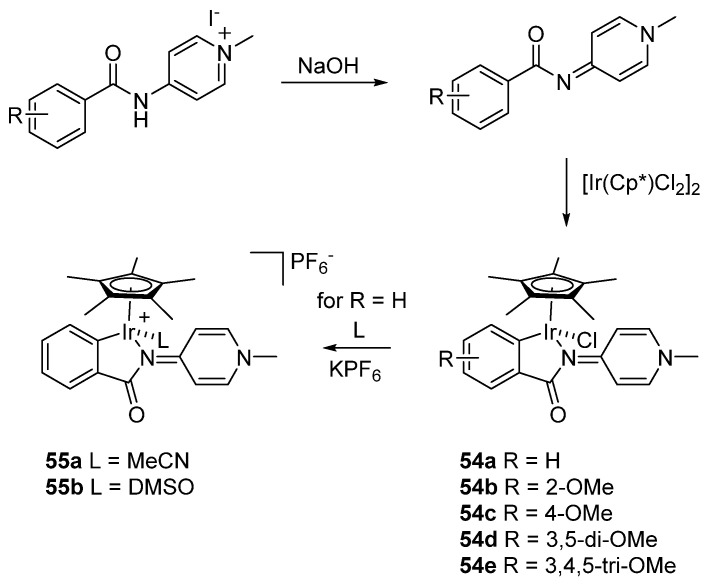
Synthesis of *N*-methyl-1,4-dihydropyridylene substituted iridacycles **54a**–**e** and **55a**,**b**.

Albrecht and coworkers tried to increase the electron donicity of the amine ligand in the iridacycles by introducing an *N*-methyl-1,4-dihydropyridylene substituent on the nitrogen atom (Scheme 33) [53]. However, in doing so, the proton on the nitrogen atom that plays an important role in the outer sphere mechanism is sacrificed. As a result, these catalysts are much slower than for instance iridacycles **36**–**40** that are highly active at ambient temperature. The catalysts were tested at 1 mol.% in the TH of benzophenone using isopropanol as reductant and solvent at 82 °C, and the conversion was measured after 2, 4, and 24 h (Table 1). Since catalyst **54d** was clearly the fastest, this catalyst was used for the reduction of a small set of ketones comprising cyclohexanone, aryl-substituted acetophenones, and 2-, 3-, and 4-acetylpryridine. All substrates could be fully reduced within 1–4 h at 1 mol.% of catalyst and reflux of isopropanol.

**Scheme 34:**
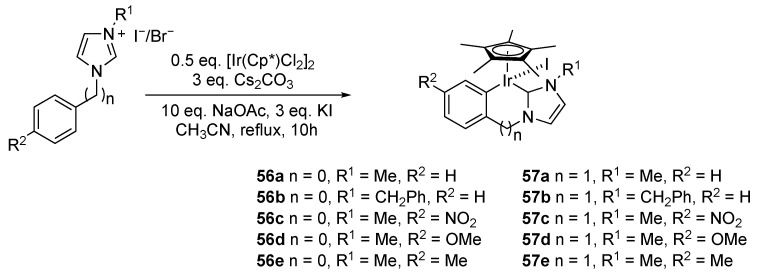
*N*-aryl and *N*-benzyl *N*-heterocyclic carbene-based iridacycles **56a**–**e** and **57a**–**e**.

Choudhoury and coworkers synthesized a series of iridacycles based on 1-aryl- or 1-benzyl-substituted *N*-heterocyclic carbenes (NHCs) (Scheme 34) [54]. Next, they investigated the yield of the TH of acetophenone as a function of the steric properties of the ligand expressed in bite-angles, as well as yaw-angles (the deviation from the ideal 180° angle between the Ir-C bond to the NHC and the angle of the NHC itself [55]). The reactions were performed in isopropanol containing 20 mol.% of KOH at 100 °C for 90 min. Although, in general, it is much better to investigate these correlations based on rates, rather than yields, it does give a first impression about a possible relationship. It turned out that reductions with catalysts **56a**–**e** were rather slow, leading to yields of 1-phenylethanol of 30–50%, whereas using catalysts **57a**–**b** and catalysts **57d**–**e** led to yields of 80–99%. Results with catalyst **57c** were also poor (45%), due to the strongly electron-withdrawing nature of the nitro group. They also constructed a Hammett plot by reducing a series of acetophenones with different *para* substituents. This resulted in a *ρ* value of 1.25, signifying the development of a negative charge on the acetophenone in the transition state. The authors assumed that, in the TS, the hydride is transferred in an inner sphere mechanism to the ketone, leading to a partial negative charge on oxygen. Ligands **57** have a larger bite-angle and a smaller yaw-angle than ligands **56**. Thus, the authors concluded that the hydricity of the iridium hydride increases with increasing bite-angle and/or decreasing yaw angle.

**Scheme 35:**
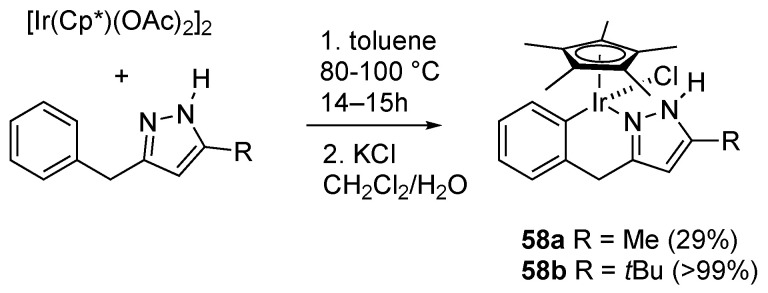
5-Alkyl-3-benzylpyrazole-based iridacycles **58a**,**b** and their performance in the transfer hydrogenation of acetophenone at 5 mol.% (yield of 1-phenylethanol after 15 h at 50 °C in isopropanol in brackets).

**Scheme 36:**
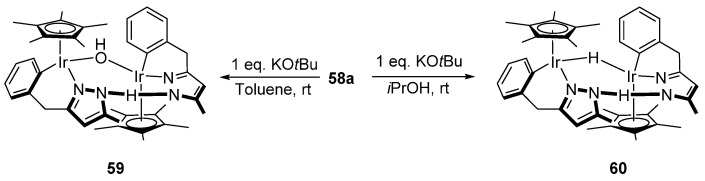
Formation of dimers **59** and **60** upon treatment of **58a** with base.

Kuwata and coworkers synthesized two new iridacycles **58** based on 5-alkyl-3-benzyl-pyrazoles (Scheme 35) [56]. Treatment of **58a** with KO*t*Bu in toluene resulted in the formation of a hydroxy-bridged dimer **59**, whereas treatment with KO*t*Bu in isopropanol led to formation of the hydride-bridged dimer **60** (Scheme 36). Interestingly, treatment of **58b** with KO*t*Bu in isopropanol led to a mixture of monomeric hydrides. This is reflected in the performance of **58a** and **58b** at 5 mol.% in the TH of acetophenone with isopropanol (1 equiv. KO*t*Bu, 50 °C, 15 h) in which **58b** is clearly the faster catalyst. The authors attributed this to the hydride bridged dimer **60** being not or less active, which would necessitate the dissociation of the dimer to obtain the active monomeric iridium hydride.

**Scheme 37:**
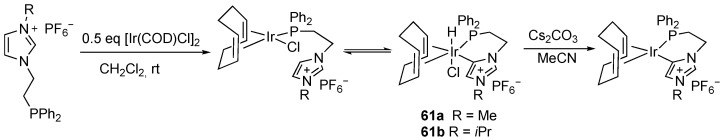
“Abnormal” *N*-heterocyclic carbene phosphine bidentate iridium complexes **61a,b**.

Zhang, Li, and coworkers prepared “abnormal” *N*-heterocyclic carbene phosphine bidentate iridium complexes **61a** and **b**, in which the iridium is bound to C5 rather than C2 of the imidazolium compound (Scheme 37) [57]. These iridacyclic complexes were found to be highly active at 0.1 mol.% in the TH of acetophenone (5 mol.% KOH, refluxing isopropanol, 5 h). In addition, catalyst **61b** was used for TH of a range of aromatic and aliphatic ketones, leading to the formation of the alcohols in good yields. The catalyst was also used for the reduction of α,β-unsaturated aldehydes and ketones (0.5 mol.% catalyst, 5 mol.% KOH, refluxing isopropanol) to yield the fully saturated alcohols in good yields.

### 3.2. Asymmetric Transfer Hydrogenation of Ketones

Ikariya and coworkers reported the first ATH of acetophenone using iridacycle catalysts **39** and **40** (Scheme 29), resulting in the production of 1-phenylethanol in 38% and 66% *ee*, respectively [49].

**Scheme 38:**
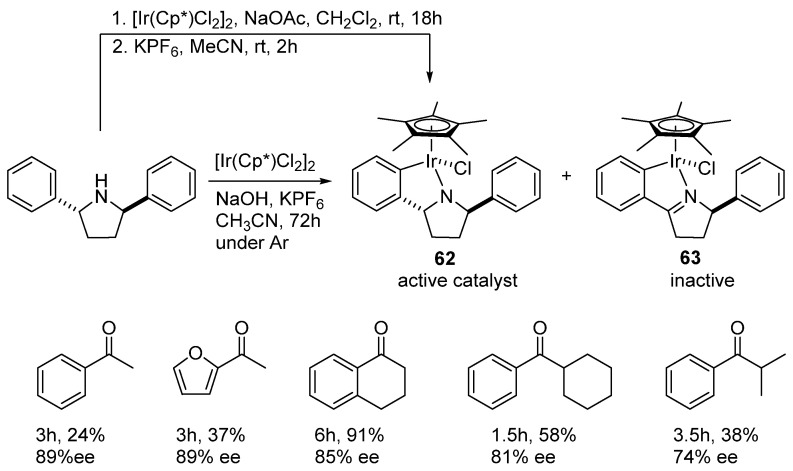
2,5-Diphenylpyrrolidine based iridacycles for the ATH of aromatic ketones (time, yield, *ee*).

Pfeffer, de Vries, and coworkers expanded upon their earlier finding that chiral ruthenacycles are excellent ATH catalysts [26], and they also synthesized iridacycles and rhodacycles to be tested in the ATH of ketones. Cyclometalation of (*R*,*R*)-2,5-diphenyl-pyrrolidine resulted in a mixture of the desired iridacycle **62** and the dehydrogenated analogous imine iridacycle **63,** which was inseparable [58]. It was possible to synthesize iridacycle **63** in pure form, and this compound turned out to be inactive as a TH catalyst. Consequently, the mixture of **62** and **63** was used for the ATH of a range of aromatic ketones (Scheme 38) [32]. Very good enantioselectivities were obtained from 74–96%.

**Scheme 39:**
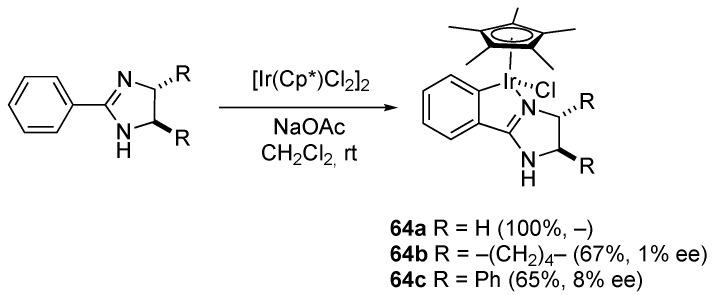
Iridacycles based on 4,5 disubstituted imidazolines and performance in the ATH of acetophenone (yield of 1-phenylethanol and *ee* after 20 h shown).

Later, Barloy, Pfeffer, and coworkers used the cyclometalation method developed by Davies [59] on 2,5-diphenylpyrrolidine and were able to prepare **62** in pure from in good yield [60]. This methodology was also used for the preparation of a set of three iridacycles **64a**–**c** which they obtained via cyclometalation of 4,5-disubstituted imidazolines. The iridacycles were tested in the ATH of acetophenone (1 mol.% of catalyst, 5 mol.% of KO*t*Bu, refluxing isopropanol). Enantioselectivities obtained were very low (Scheme 39). Conversion and enantioselectivity was measured after 4 and 20 h. The authors noted some erosion of *ee* over time.

**Scheme 40:**
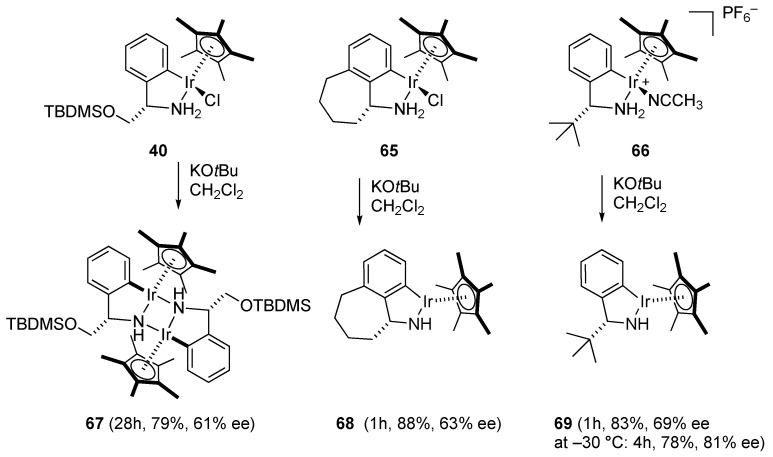
Chiral iridacycle complexes and their performance in the ATH of acetophenone (time, yield, *ee*).

Kayaki, Ikariya, and coworkers synthesized three chiral iridacycles by cyclometalation of the primary amines [61]. Treatment of **40** with KO*t*Bu in CH_2_Cl_2_ resulted in the formation of a dimeric complex **67** in which the two 16e complexes are fused by a four-membered ring formed by the bonds between iridium and the nitrogen atom of the second complex. However, treatment of **65** or **66** with base in the same way allowed isolation of the monomeric 16e complexes **68** and **69** (Scheme 40). The complexes **67**–**69** were tested in the ATH of acetophenone (0.1 mol.%, no base, isopropanol, 30 °C). Catalysts **68** and **69** were clearly faster than the dimeric catalyst **67**. With all three catalysts, decent enantioselectivities were achieved. The authors noted erosion of *ee* over time. In an experiment at S/C = 200, reactions were complete within 1 h; if the reactions were left for longer periods, the *ee* gradually diminished to reach close to zero after 20 h. Indeed, Feringa, de Vries and coworkers earlier established that iridacycles are excellent alcohol racemization catalysts [45].

**Scheme 41:**
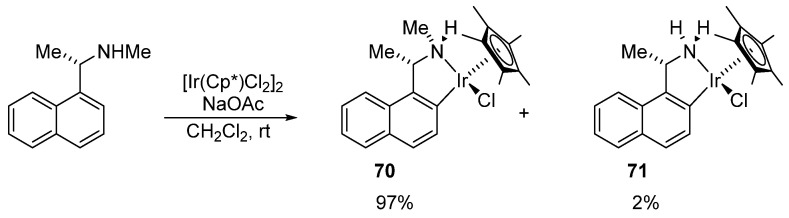
Iridacycles obtained by cyclometalation of (*S*)-*N*-methyl-*N*-naphthy-1-yl-ethylamine.

Leung and coworkers examined the cyclometalation of (*S*)-*N*-methyl-*N*-naphthy-1-yl-ethylamine using [Ir(Cp*)Cl_2_]_2_ and NaOAc in CH_2_Cl_2_ at RT. Surprisingly, they found a mixture of the expected iridacycle **70** and a small amount of the analogous demethylated iridacycle **71** (Scheme 41) [62]. They managed to isolate both compounds in pure form. Iridacycle **70** was investigated extensively. The complex is a pure diastereomer and, even after treatment with base followed by treatment with HCl, the same single diastereomer was obtained. Both **70** and **71** were examined as catalysts in the ATH of acetophenone and found to show very similar behavior in terms of rate and enantioselectivity. At −15 °C with both catalysts (2 mol.%), activated by KO*t*Bu, over 90% conversion was reached after 30 min and the *ee* of the product alcohol was 60% in both cases. Catalyst **70** was also tested at −30 °C and, in this case, 97% conversion was reached after 1.5 h, and the product had an *ee* of 69%. At room temperature, the reaction catalyzed by **70** was finished within 15 min and the product was obtained with 52% *ee*. The same catalyst was capable of reducing acetophenone without activation by base at RT, but the reaction was rather slow and needed 97 h to reach 75% conversion and an *ee* of 58%.

**Scheme 42:**
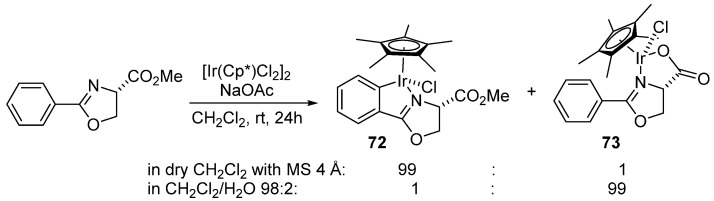
Products of the cyclometalation of methyl (*S*)-2-phenyl-oxazoline-4-carboxylate.

**Figure 3:**
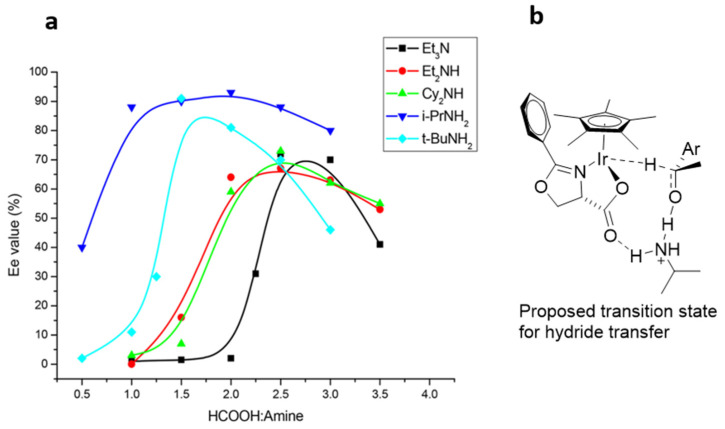
(**a**) Effect of the structure of base and the formic acid/base ratio on the enantioselectivity of the ATH of *p*-nitro-acetophenone catalyzed by **73**. (**b**) Proposal for the transition state of the hydride transfer (**a**), reprinted with permission from [63]; copyright 2018 American Chemical Society.

Chen, Xiao, and coworkers examined the cyclometalation of methyl (*S*)-2-phenyl-oxazoline-4-carboxylate [63]. Depending on the amount of water in the solvent CH_2_Cl_2_, they found varying amounts of complexes **72** and **73** (Scheme 42). By performing the reaction in ultra-dry solvent in the presence of MS 4 Å, it was possible to obtain **72** in 99% purity. On the other hand, addition of 2 vol.% of water to the solvent resulted in almost exclusive formation of complex **73**. Both complexes were tested in the ATH of *p*-nitro-acetophenone using the HCO_2_H/Et_3_N azeotropic mixture (5:2) as reductant in various solvents. Reactions catalyzed by iridacycle **72** were slow in all solvents and afforded the product with very low enantioselectivity. In contrast, use of complex **73** resulted in a fast reaction, and, in CH_2_Cl_2_, the product was obtained with 73% *ee*. Next, the authors examined the influence of the structure of the base and the formic acid/base ratio. Best results were obtained using *i*-PrNH_2_ as base and a formic acid/base ratio of 2.0 (Figure 3a). The authors explained this by assuming a transition state for the hydride transfer in which the protonated base plays the role of proton shuttle (Figure 3b). The authors used these optimized conditions for the reduction of a range of 20 different substituted acetophenones and other aryl ketones with *ee*s ranging from 92–99%.

**Scheme 43:**
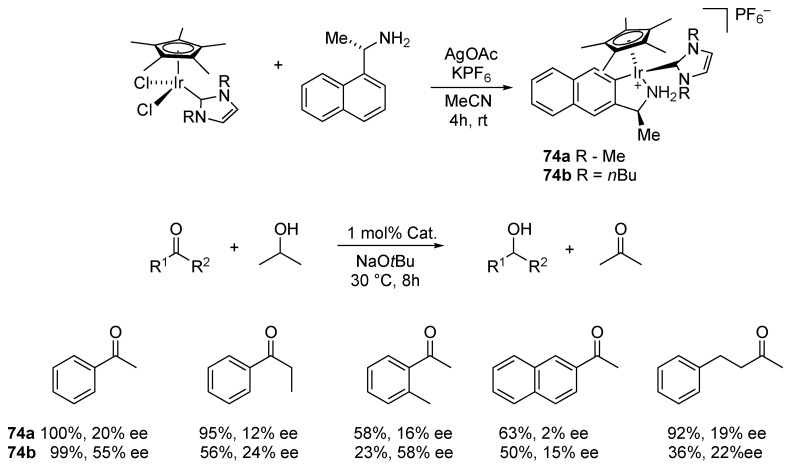
Cationic NHC-ligated iridacycles as catalysts for the ATH of aromatic ketones.

**Figure 4:**
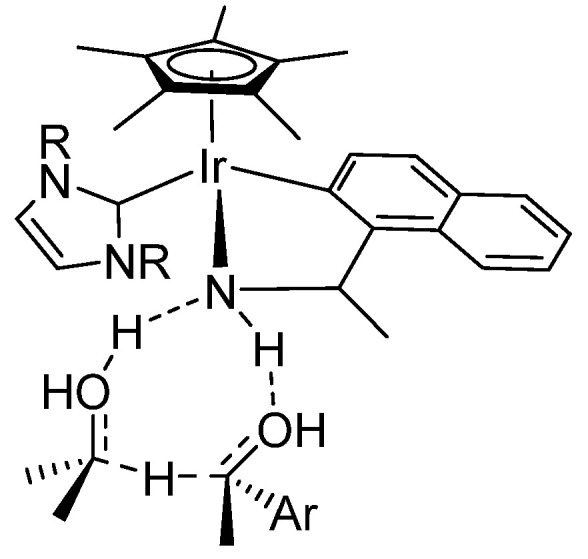
Transition state for MPV mechanism in ATH catalyzed by **74a** and **74b**.

Baya, Mata, and coworkers prepared two NHC-ligated iridacycles **74a** and **74b** via reaction of Ir(Cp*)(NHC)Cl_2_ with (*R*)-1-naphth-1-yl-ethylamine under the influence of AgOAc and KPF_6_ [64]. After treatment with base, which presumably deprotonates the amine group to from the amide complex. These complexes were active as ATH catalysts. A small set of four aromatic and one aliphatic ketone was reduced to the alcohols with *ee*s between 2% and 58% (Scheme 43). This activity is somewhat surprising as no ligand position is available for the formation of a hydride. On the basis of DFT calculations, the authors proposed a Meerwein–Ponndorf–Verley type mechanism in which the amide group deprotonates isopropanol, which transfers its hydride to the ketone, which is protonated by the remaining proton of the amide ligand. The proposed transition state is depicted in Figure 4.

### 3.3. Oxidation and Dehydrogenation of Alcohols to Aldehydes, Ketones, Carboxylic Acids, or Esters

**Scheme 44:**
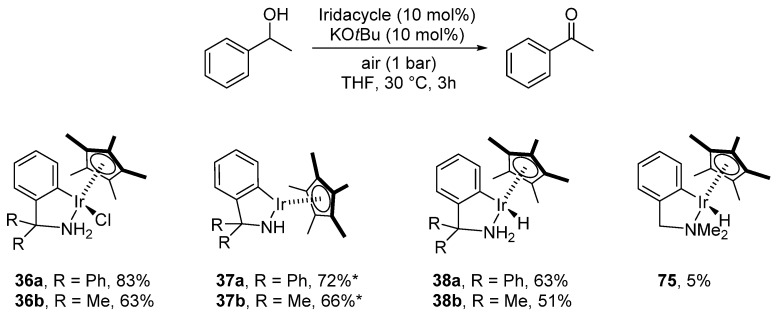
Performance of iridacycles in the aerobic oxidation of 1-phenylethanol (yield of acetophenone in brackets; * no base was used).

Ikariya and coworkers investigated the iridacycles **36**–**38** and iridacycle **75** as catalyst for the oxidation of 1-phenylethanol with air in THF at room temperature (Scheme 44) [65]. All catalysts with the exception of **75** were quite effective, although 10 mol.% of catalyst was needed. The lack of activity with **75** can be explained by assuming an iridium hydride amine/iridium amide redox couple is operative in this reaction. Next, the catalyst **37a** was used for the oxidation of a series of substituted 1-phenyl-ethanols, benzhydrol, and 4-phenyl-butan-2-ol in good yield. Cyclohexenol was oxidized to cyclohexanone in 47% yield. Oxidation of hydroxyketones was slow, and benzil was formed in only 10% yield from benzoin. Similarly, methyl mandelate could not be oxidized. Oxidation of diols to the hydroxyketones was possible in yields from 31–34%. Benzyl alcohols were oxidized to the benzyl benzoates in yields from 62–72% using catalyst **36a**. Oxidative esterification of aliphatic alcohols was more problematic and gave the products in 45–46% yields. The authors assumed a mechanism in which the dehydrogenation of the alcohol takes place concertedly on the 16 electron iridacycle **37a** to give the protonated hydride complex **38a**. The hydride complex next reacts with oxygen to reform the complex **37a**.

The chiral iridacycle **76,** as well as a number of iridium complexes based on chiral *N*-sulfonated diamines such as **77,** was tested in the kinetic resolution of secondary alcohols via oxidation [66]. Iridacycle **76** (10 mol.%, 1M substrate concentration in THF)) was used for the oxidation of 1-phenylethanol at room temperature, and the reaction was stopped after 4 h when 48% of alcohol was unconverted (Table 2). Lowering the concentration of the substrate to 0.1 M improved the *ee* to 48%, but now 75 h was needed to reach the 52% conversion. In the end, the catalysts based on the sulfonated diamines performed better. With catalyst **77**, it was possible to reach 98% *ee* after 52% conversion at 0.2 M substrate concentration, whereas, at 1.0 M, it was possible to reach 84% *ee* after 56% conversion. The authors gave no explanation for the concentration effect.

**Scheme 45:**
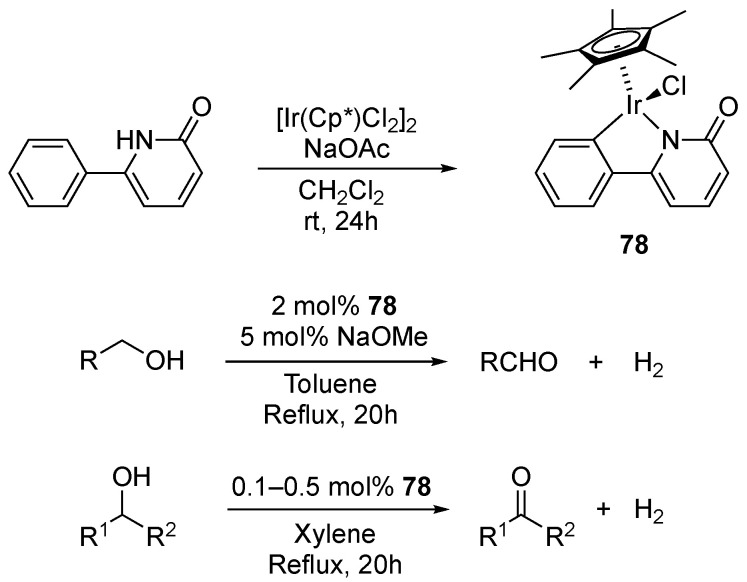
Iridacycle-catalyzed dehydrogenation of primary and secondary alcohols.

Interestingly, oxygen is not even necessary to oxidize alcohols. It is also possible to use an iridacycle as a dehydrogenation catalyst. Fujita, Yamaguchi, and coworkers examined the use of benzopyridone-based iridacycle **78** in the dehydrogenation of benzyl alcohols (Scheme 45) [67]. The products were obtained in excellent yields, i.e., around 90% yield when no substituents or electron-donating substituents were present. For benzyl alcohols carrying electron-withdrawing substituents, it was necessary to raise the temperature by carrying out the reaction in refluxing xylene and using Na_2_CO_3_ as base. Even aliphatic alcohols such as cyclohexylmethanol and 1-octanol could be dehydrogenated to the aldehydes by using 5 mol.% of catalyst with refluxing xylene as solvent in 62% and 46% yield, respectively. The dehydrogenation of secondary alcohols was even more facile and could be performed using between 0.1 and 0.5 mol.% of catalyst in refluxing xylene without any base yielding the ketones in yields varying from 90–100%. The authors assumed that an inner-sphere mechanism is operative.

**Scheme 46:**
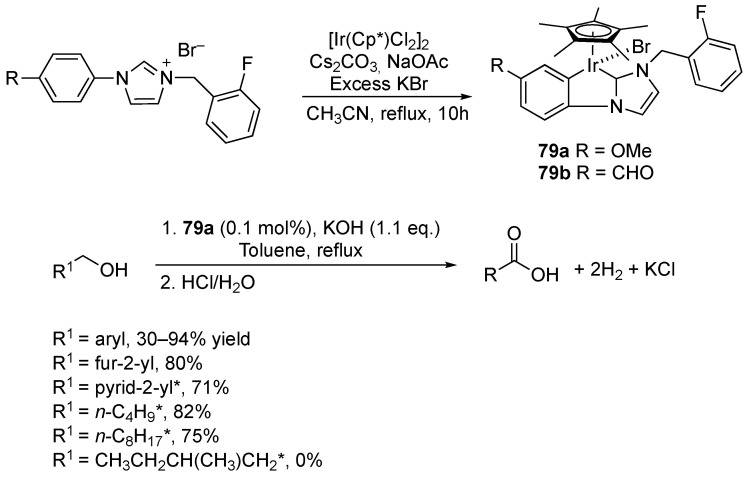
Iridacycle-catalyzed dehydrogenation of alcohols to acids (* isolated as potassium salt).

Das and coworkers investigated the use of iridacycles **79a** and **b** in the dehydrogenation of alcohols to the carboxylic acids in refluxing toluene [68]. The reaction needs a stoichiometric base, and KOH was found to perform best. Use of weaker bases such as K_2_CO_3_ and Cs_2_CO_3_ led to no reaction. Catalyst **79a** was found to perform better than **79b**, presumably because of the electron-donating substituent of **79a**. Instability of the aldehyde group of **79b** turned out not to be the reason for the lower reactivity. A range of benzyl alcohols was synthesized in this manner in yields between 80% and 90% (Scheme 46). Electron-withdrawing substituents and *ortho*-substituents reduced the yields of the acids. The acids were isolated by acidification at the end of the reaction. Some mechanistic research was performed. The authors were able to measure the production of 2 equiv. of hydrogen at the end of the reaction. The first step is the production of the aldehyde. This intermediate can be converted into the acid via two different pathways: either via Cannizzaro reaction or via the catalyzed dehydrogenation of the hydrate of the aldehyde. The authors were able to prove that both mechanisms were operative. The catalyst was highly active and could be used at 0.1 mol%. It was also reused three times with any discernable loss in yield. A maximum of 5000 turnovers was reached in the oxidation of benzyl alcohol.

Surprisingly, no Oppenauer-type oxidations were reported with iridacycles, although there is one report on the use of an iridium-pincer complex (outside the scope of the review) [69].

### 3.4. Transfer Hydrogenation of Imines and Reductive Amination

**Scheme 47:**
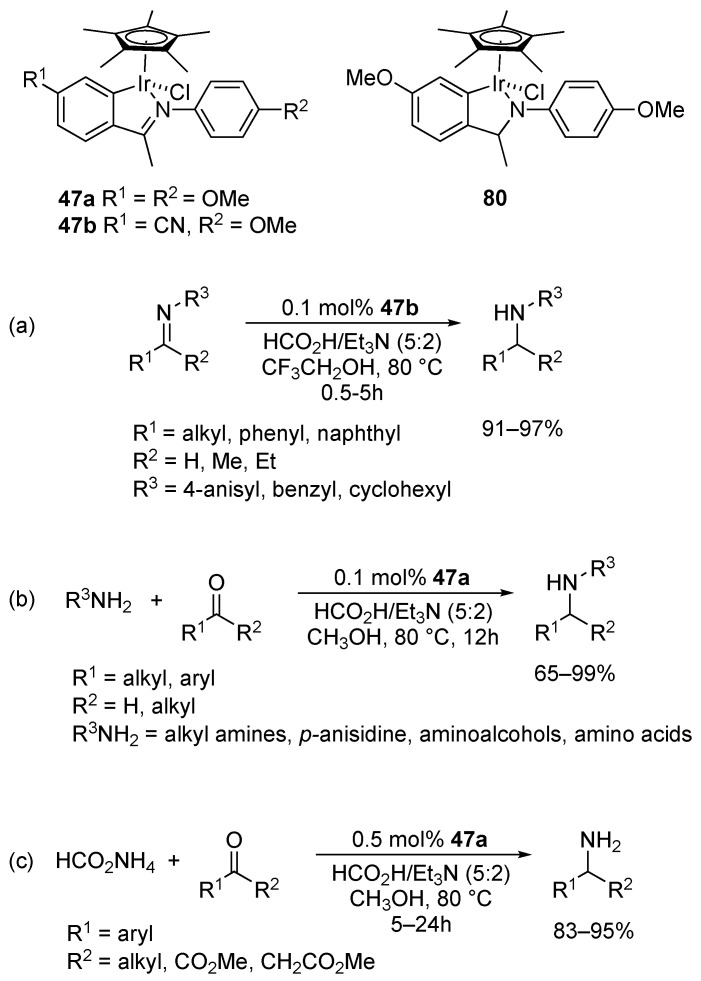
Iridacycle-catalyzed imine hydrogenation (**a**) and reductive amination of ketones to secondary amines (**b**) and to primary amines (**c**).

**Scheme 48:**
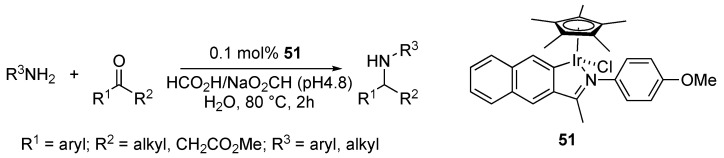
On-water iridacycle-catalyzed reductive TH.

Xiao and coworkers discovered the activity of the acetophenone imine-based iridacycles by accident in the course of investigating the reactivity of iridium complexes based on tosylated diamines when they tested the activity of the catalyst precursor in the hydrogenation of 2-heptanone anisidylimine [70]. They were able to isolate the corresponding iridacycle from the reaction mixture. Next, they compared the activity of premade iridacycles **47a**,**b** and **80** in the TH of the same substrate at 80 °C using HCO_2_H/Et_3_N (5:2) in trifluoroethanol as reductant. The three premade iridacycles were much faster than the in situ formed iridacycle from [Ir(Cp*)Cl_2_]_2_ with **47b** > **47a** >> **80**. The poor reactivity of the iridacycle based on the amine **80** is surprising since Pfeffer and de Vries found quite the reverse in the TH of ketones with isopropanol where the imine-based catalyst was inactive [32]. The authors did not offer any explanation. The TH using catalyst **47b** is extremely fast with an initial TOF of 1.9 × 10^4^. The authors assumed that a direct hydride transfer from the iridium hydride to the protonated imine takes place. A range of imine substrates was reduced in excellent yield using only 0.1 mol.% of catalyst **47b** (Scheme 47a). Catalyst **47a** was used for the direct reductive amination via TH (Scheme 47b). Here, the use of trifluoroethanol was not necessary and methanol could be used. Depending on the type of substrate, 0.1 or 0.5 mol.% of catalyst was used. It was even possible to use complex **47a** as catalyst for a direct reductive amination using ammonium formate as the amine donor (Scheme 47c). In a later publication, the direct reductive amination was examined further, and seven iridacycles were screened [71]. Complex **51** (see Scheme 48 for structure) gave the best results, and a large number of aromatic ketones were converted into the primary amines with excellent yields using only 0.1 mol.% of catalyst.

The mechanism of the imine TH was investigated using kinetics, NMR studies, and DFT calculations [72]. It turned out that hydride formation from the iridacycle formate complex is the rate-determining step. The hydride transfer occurs directly on the protonated imine without the imine binding to the metal.

The same group found that the iridacycle-catalyzed reductive amination of aldehydes and ketones with amines via TH can also be performed using formate salts in water in an “on-water” procedure as the catalysts are not soluble in water [73]. This reaction is again crucially dependent on the pH of the water with the lower pH preferred since this not only catalyzes the imine formation, but the acid also protonates the imine, which allows the transfer of hydride from the iridacycle. At the optimum pH, the reduction of the ketone to the alcohol is a major side-reaction, which could be mitigated by working at a somewhat higher pH. In the end, pH 4.8 was chosen as compromise as formation of alcohol is largely suppressed at this pH. Seven iridacycles based on *para*-substituted acetophenone-*N*-phenylimine were screened, and best results were obtained with catalyst **51**. Interestingly, performance of the TH in methanol or DMF led to homogeneous systems, but the rate of the reactions in these solvents was substantially lower, supporting the “on-water” effect. A range of aromatic ketones was subjected to reductive amination with anilines of alkylamines at 0.1 mol.% of catalyst with a sodium formate/formic acid-buffered aqueous solution at 80 °C (Scheme 48). Yields were generally above 90% with substituted anilines and somewhat lower with alkylamines.

**Scheme 49:**
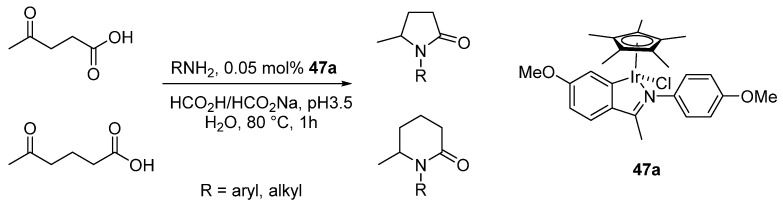
Iridacycle-catalyzed reductive amination of keto acids to form pyrrolidinones and piperidones.

Xiao and coworkers also used the reductive amination technology they developed for the formation of *N*-aryl and *N*-alkyl 5-methyl-pyrrolidinones from levulinic acid and for the formation of *N*-aryl and *N*-alkyl 6-methyl-piperidones from 5-oxo-hexanoic acid (Scheme 49) [74]. The reaction was performed using anilines of alkylamines in an aqueous formic acid/sodium formate buffer at pH 3.5 and 80 °C. With most substrates, an S/C ratio of 2000 could be used, and the reaction was finished within 1 h. More sluggish substrates needed a S/C of 200–500. Yields of the products were excellent.

Albrecht and coworkers tested the iridacycles of Scheme 33 in the transfer hydrogenation of benzaldehyde *N*-phenylimine in the presence of catalytic KOH in refluxing isopropanol. Although the five complexes performed quite differently in the TH of acetophenone, in the TH of this substrate, all five catalysts were equally fast. In the end, catalyst **54b** was selected for the TH of a small set of *N*-aryl and *N*-alkyl benzaldehyde imines in good yields. Reduction of an acetophenone based imine was very slow [53].

### 3.5. Asymmetric Hydrogenation of Imines

**Scheme 50:**
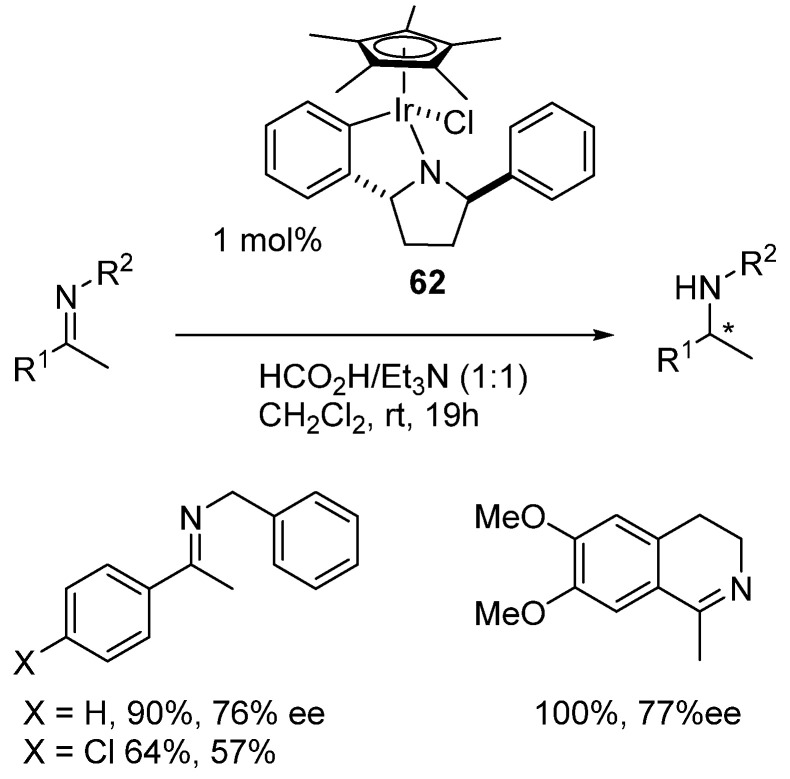
ATH of imines using iridacycle **62** as catalyst.

Pfeffer, de Vries, and coworkers used iridacycle **62** for the ATH of three imines using a 1:1 mixture of formic acid and triethylamine in dichloromethane at room temperature (Scheme 50) [32]. Yields were good and enantioselectivities between 57% and 77% were obtained. The authors noted the importance of working under strict exclusion of water to prevent hydrolysis of the imine. This was achieved by adding dry Et_3_N to the azeotropic mixture of formic acid and triethyl amine (5:2) to achieve a 1:1 ratio.

**Scheme 51:**
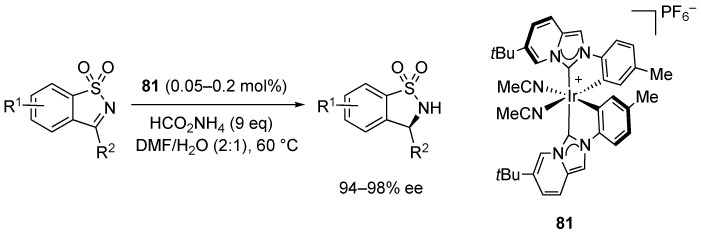
Chiral-only-at-metal iridacycle catalyzed enantioselective TH of sulfonylimines.

Gong, Meggers, and coworkers earlier developed a chiral-at-metal iridacycle, which they applied for the ATH of cyclic sulfonylimines using ammonium formate as a reducing agent (Scheme 51) [75]. The catalyst was highly active and could be used at 0.05–0.2 mol.% at 60 °C. Yields of the products were excellent and enantioselectivities were between 94% and 98%. The authors performed some mechanistic investigations and found that the first event is the formation of a complex between **81** and NH_3_. This complex is next converted to the hydride complex Ir(C-N)_2_(NH_3_)H. It was proposed that the ammonium group plays a major role in the positioning of the substrate resulting in very high enantioselectivities.

**Scheme 52:**
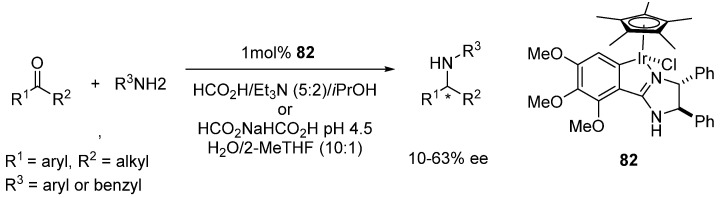
Enantioselective reductive amination catalyzed by iridacycle **82**.

Xiao and coworkers screened 15 different chiral iridacycles based on 4,5-diaryl-substituted 2-phenyl-oxazolines and imidazolines in the enantioselective reductive amination of acetophenone with *p*-anisidine using the formic acid triethylamine azeotrope in isopropanol as reductant at ambient temperature [76]. From this screening, iridacycle **82** came out as the best one, inducing the highest enantioselectivity. This catalyst was then used in enantioselective direct reductive amination of aromatic ketones and anilines or benzyl amines (Scheme 52). As reductant, the azeotropic formic acid/triethylamine mixture in isopropanol was used. In cases where the yield was low, it was possible to improve this by using a sodium formate/formic acid buffer in water with 10 vol.% of 2-MeTHF as cosolvent, to increase solubility. However, this did not affect the enantioselectivity.

### 3.6. Transfer Hydrogenation of Heterocycles

**Scheme 53:**
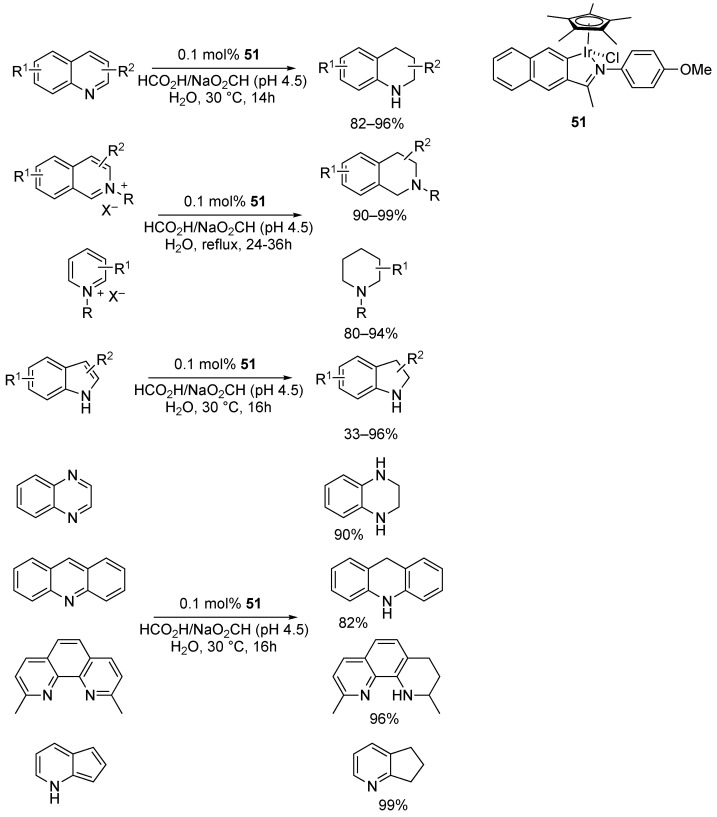
Iridacycle-catalyzed TH of nitrogen heterocycles.

Xiao and coworkers examined the transfer hydrogenation of nitrogen heterocycles using three different catalysts: [Ir(COD)(NHC)(PPh_3_)]BF_4_, [Rh(Cp*)(Ts-DPEA)]OTf, and iridacycle **51**. Whereas hardly any reactivity was observed with the first two catalysts, the iridacycle performed very well, and quinolines (26 examples) could be reduced to the 1,2,3,4-tetrahydroquinolines in excellent yields using only 0.1 mol.% of **51** in an aqueous sodium formate/formic acid buffer at pH4.5 at 30 °C (Scheme 53). This method did not work on isoquinoline or on 2-phenylpyridine. Nevertheless, it was possible to reduce these classes of compounds after quaternization, although it was necessary, in this case, to perform the reaction at reflux. In this way, six isoquinolinium compounds and 10 pyridinium compounds were reduced. Interestingly, pyridinium salts carrying electron donating substituents in the 3- or 4-position were reduced to the 1,2,3,5-tetrahydropyridines, whereas those with electron withdrawing substituents were fully reduced to the piperidines.

This can be explained by assuming that, in the latter case, the first step is a 1,4-reduction, whereas, in the former case, it is a 1,2-reduction. Whereas unprotected indoles are problematic substrates for homogeneous hydrogenation, transfer hydrogenation of indoles proceeded effortlessly using 0.1 mol.% of **51** at 30 °C. The authors tested seven substrates, of which only 2-phenyl-indole could not be reduced. Quinoxaline was converted to 1-formyl-1,2,3,4-tetrahydroquinoxaline. Acridine was reduced to dibenzopiperidine. 2,9-Dimethyl-1,10-phenanthroline was reduced to the 1,2,3,4-tetrahydro-product exclusively. 1*H*-Cyclopenta[b]pyridine was selectively reduced on the cyclopentadiene part to yield cyclopentanopyridine.

**Scheme 54:**
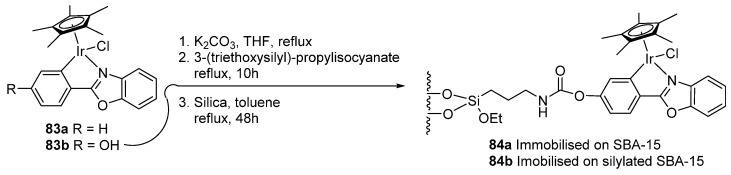
Immobilization of iridacycle catalysts **83a**,**b**.

Hou and coworkers immobilized the iridacycle which they prepared from 4-(benzo[d]oxazol-2-yl)phenol **83b** on mesoporous silica (SBA-15 and on silylated SBA-15) (Scheme 54) [77]. The resulting catalyst **84a** was used for the TH of quinolines. If the formic acid/sodium formate buffer system that was developed by Xiao and coworkers was used at 80 °C, isoquinoline was reduced to the 1,2,3,4-tetrahydroquinoline, which was formylated in excellent yield. If formic acid was used in water or in the presence of a sodium acetate/acetic acid mixture, no formylation occurred. There was no difference in performance between **84a** and **84b**. The authors compared the rate of the immobilized catalyst **84a** with that of **83a**, which is an unfair comparison as Xiao and coworkers showed that the methoxy substituent leads to faster reaction. The immobilized catalyst had a faster onset; however, in the end, both catalysts needed the same time for complete conversion of the substrate. The immobilized catalyst could be reused 12 times in the reduction/formylation reaction with only 20% loss in yield after the 12th time. Nevertheless, the usefulness of the immobilization remains questionable in view of the increased cost and the loss of activity over time [78].

**Scheme 55:**
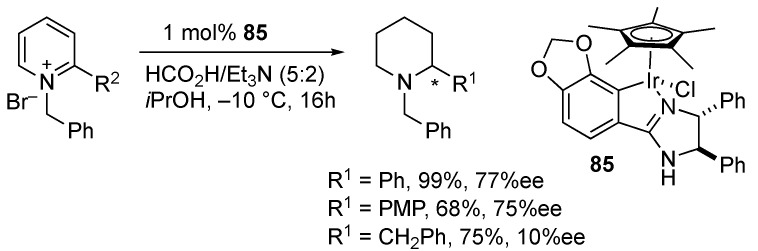
Asymmetric transfer hydrogenation of pyridinium salts.

Xiao and coworkers tested 15 different chiral iridacycles based on 4,5-diaryl-substituted 2-phenyl-oxazolines and imidazolines in the enantioselective hydrogenation of *N*-benzyl 2-phenylpyridinium bromide using the azeotropic mixture of formic acid and triethylamine in isopropanol as solvent at room temperature [76]. Best results (55% *ee*) were obtained with iridacycle **85**. This could be further improved to 77% at −10 °C. Reduction of two more 2-substituted pyridines was reported (Scheme 55).

### 3.7. Dehydrogenation of Heterocycles

**Scheme 56:**
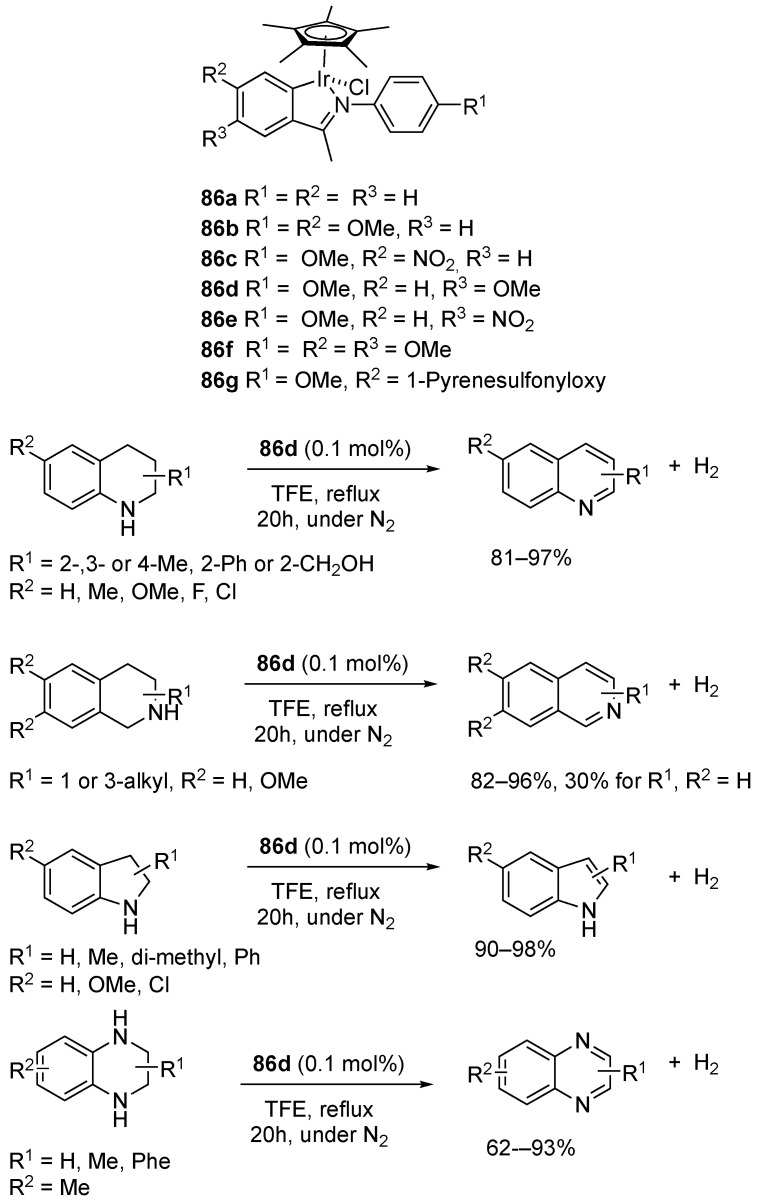
Iridacycle-catalyzed acceptorless dehydrogenation of nitrogen heterocycles.

Xiao and coworkers examined the acceptorless dehydrogenation of 1,2,3,4-tetrahydroquinolines using iridacycles **86a**–**f** as catalyst (Scheme 56). From the first screen, complex **86d** achieved the highest conversion. A solvent screen uncovered a strong solvent dependence. Of all the tested solvents, i.e., polar, nonpolar, and protic solvents, trifluoroethanol (TFE) emerged as the only solvent that resulted in very good yields of the dehydrogenated products [79]. The authors were able to dehydrogenate a range of substituted 1,2,4-tetrahydroquinolines. The dehydrogenation is surprisingly selective for nitrogen compounds; a hydroxymethyl substituent survived the reaction unchanged. Tetrahydroisoquinolines could also be dehydrogenated in excellent yields, with the exception of the unsubstituted compound, which was converted to isoquinoline in only 30% yield. Indolines and 1,2,3,4-tetrahydroquinoxalines were also dehydrogenated to the indoles and quinoxalines, respectively, in good yields.

Chen, Lu, and coworkers decided to immobilize the iridacycle catalyst. Thus, they synthesized pyrene-tagged iridacycle **86g** (Scheme 56) by reacting the phenol group of the iridacycle with 1-pyrenylsulfonylchloride. This compound was immobilized on carbon nanotubes, and the immobilized catalyst was used for the dehydrogenation of indolines [80]. They had to use a mixture trifluoroethanol and water to assure the strong attachment between the pyrene and the carbon nanotubes. It was necessary to perform the reaction at 1 mol.% of catalyst, which is 10-fold more than the homogeneous catalysts from Xiao, which again shows the futility of an immobilization approach. In addition, the reaction temperature had to be raised to 100 °C. The catalyst could be reused a number of times with some loss of activity after each run. The catalyst was compared with the homogeneous analogue, albeit at high temperature and high catalyst loading; hence, not surprisingly, both catalysts led to good yields. This comparison needs to be done on rate rather than yield.

### 3.8. Borrowing Hydrogen Reactions

**Scheme 57:**
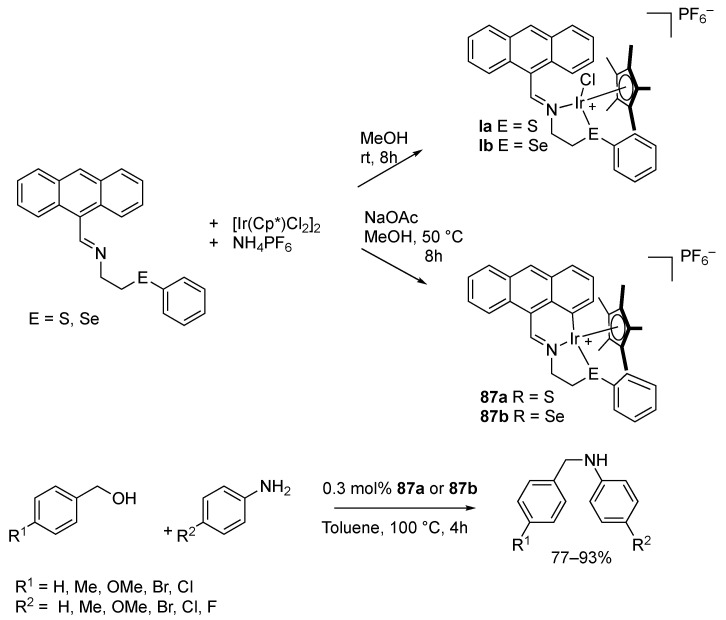
Iridacycle-catalyzed borrowing hydrogen alkylation of anilines with benzyl alcohols.

Singh and coworkers reacted a bidentate imino thio (or seleno) ligand with [Ir(Cp*)Cl_2_]_2_ in methanol at room temperature and isolated the complexes **Ia** and **Ib** in good yields (Scheme 57) [81]. If the same reaction was performed at 50 °C in the presence of sodium acetate, the iridacycles **87a** and **87b** were obtained. All four complexes performed well in the TH of acetophenones and benzaldehydes. Next, the authors examined their application in the borrowing hydrogen alkylation of anilines with benzyl alcohol. In these reactions, the alcohol is first dehydrogenated to the aldehyde, which reacts with the aniline to form the imine, which is reduced with the hydrogen equivalents produced in the first dehydrogenation. The products were obtained in excellent yields. The iridacycles gave slightly lower yields than the complexes **Ia** and **Ib**. In addition, the sulfur-containing complexes performed slightly better than the selenium-containing complexes.

**Scheme 58:**
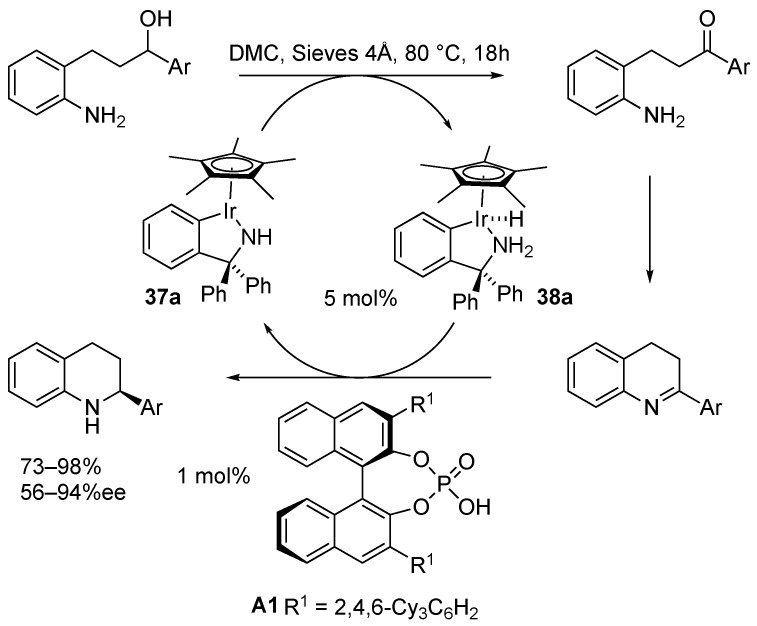
Enantioselective synthesis of 2-aryl-susbtituted tetrahydroquinolines via a borrowing hydrogen reaction.

Zhao and coworkers examined the enantioselective synthesis of 2-aryl-substituted 1,2,3,4-tetrahydroquinolines via a borrowing hydrogen reaction from racemic 1-aryl-substituted 4-(2-aminophenyl)propanols [82]. In this reaction, the alcohol is first dehydrogenated to the ketone, which reacts with the aniline to form the 3,4-didhydroquinoline, which is enantioselectively reduced to the tetrahydroquinoline (Scheme 58). They tested a range of nine chiral and nonchiral iridium catalysts in combination with a chiral BINOL- or SPINOL-based phosphoric acid. Highest enantioselectivities were obtained with a combination of iridacycle **37a** and phosphoric acid **A1**. A range of aryl-substituted starting materials were converted in very good yield and excellent *ee*s. Only the 2-furyl compound was isolated in somewhat lower yield (73%) and had lower *ee* (56%). The cyclohexyl compound was also obtained in 95% yield and 80% *ee*.

**Scheme 59:**
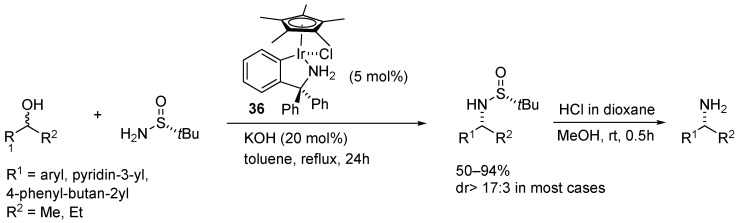
Diastereoselective synthesis of primary amines using iridacycle-catalyzed borrowing hydrogen chemistry.

Zhang, Xia, and coworkers also used the borrowing hydrogen technology in combination with Ikariya iridacycle **36** for the diastereoselective conversion of racemic alcohols to amines [83]. Reaction of the racemic alcohol with *t*-butanesulfinamide, catalyzed by iridacycle **36** activated by 20 mol.% KOH resulted in the formation of the *t*-butylsulfinylamines with diastereoselectivites that were mostly better than 17:1 and, in many cases, better than 19:1 (Scheme 59). The sulfinyl protecting group was easily removed by acidic hydrolysis in 90% yield.

### 3.9. Racemization of Alcohols and Amines

**Scheme 60:**
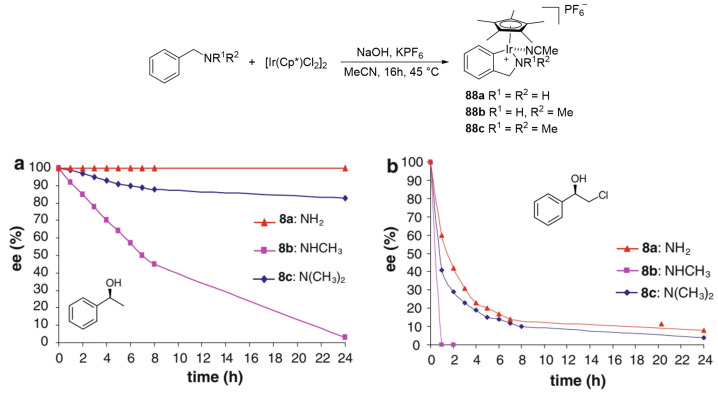
Use of iridacycles **88a**–**c** as alcohol racemization catalysts for 1-phenylethanol (**a**) and 1,phenyl-2-chloro-ethanol (**b**) at room temperature (graphs copied from [31].

Feringa, de Vries, and coworkers synthesized three benzylamine-based iridacycles **88a–c**, which differed in the substitution on the amino group [31,45]. They were tested in the racemization of enantiopure 1-phenylethanol, as well as enantiopure 1-phenyl-2-chloroethanol, at room temperature (Scheme 60). With both substrates, the catalyst based on the secondary amine **88b** performed much better than the other two catalysts (Scheme 60). Until today, this is one of the fastest racemization catalysts known.

Iridacycle **88b** is also active in the presence of water, which allowed its use as racemization catalyst in the dynamic kinetic resolution (DKR) of racemic halohydrins [46]. The enzyme haloalcohol dehalogenase catalyzes the interconversion of halohydrins and epoxides. The enzyme selectively converts the *R*-enantiomer to the analogous epoxide. In the presence of an alcohol racemization catalyst, it should be possible to racemize the remaining *S*-enantiomer, allowing full conversion of the racemate to the enantiopure epoxide. Unfortunately, the enzyme and catalyst **88b** were mutually incompatible. Both were deactivated in each other’s presence, even in a two-phase aqueous/organic system.

**Scheme 61:**
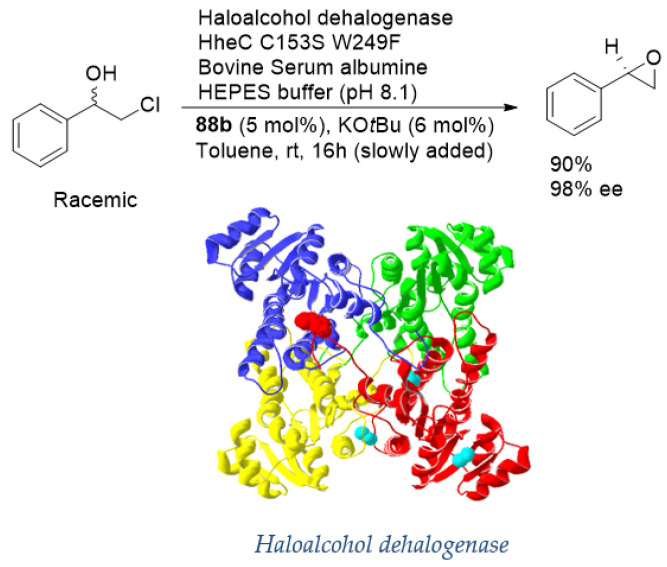
Dynamic kinetic resolution of halohydrins using iridacycle **88b** as racemization catalyst.

This problem could be solved by adding serum albumin, a protein that preferentially resides at the aqueous organic interphase and, in this way, creates a barrier between the two catalysts. In addition, the solution of **88b** was slowly added over time to mitigate its decomposition. In this way, it was possible to convert 1-phenyl-2-chloroethanol to (*R*)-styrene oxide in 90% yield and 98% *ee* (Scheme 61) [46].

Catalyst **88b** was further tested on a range of other aromatic and aliphatic alcohols. With all methyl alcohols, small amounts of the ketones were formed as side-product, which is an indication of the fact that the ketone reduction is the rate-determining step in these racemizations.

**Scheme 62:**
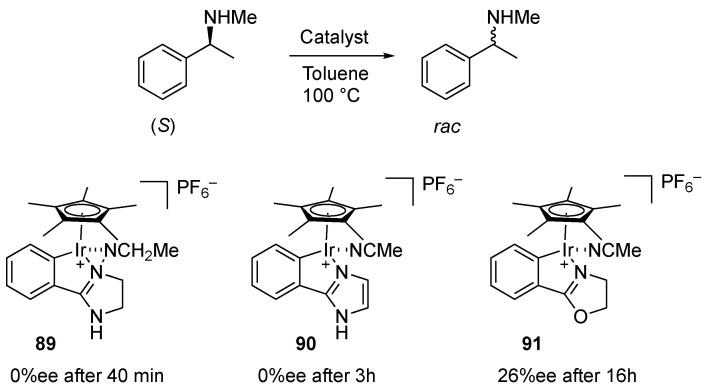
Iridacycle catalysts for the racemization of secondary amines.

They also investigated the use of these iridacycles for the racemization of secondary amine [45]. Catalysts **88a**–**c** were efficient in the racemization of (*S*)-α-methyl-*N*-methylbenzylamine, since complete racemization was observed within 8 h in chlorobenzene and within 16 h in toluene at 100 °C when using 2 mol.% of catalyst. However, the most active catalyst **88b** possessing a secondary amine as the ligand seemed to be deactivated under the reaction conditions. It was found that this was due to dehydrogenation of the ligand to the imine. Consequently, the authors designed three new iridacycles **89**–**91** for use as amine racemization catalyst. Catalyst **89** could possibly still be oxidized and, hence, catalyst **90** was also synthesized to establish if this catalyst is still active. Catalyst **91** was synthesized to establish the importance of the presence of the NH group. Catalyst **89** was capable of racemizing the secondary amine completely in 40 min; within 5 min, the *ee* was already reduced to 50%. For catalyst **90**, these values were 3 h and 20 min. Catalyst **91** was still capable of racemizing the secondary amine, but needed 16 h to reach 26% *ee*, establishing the importance of the NH functionality (Scheme 62). Use of chlorobenzene as solvent increased the rate of the racemization, and catalyst **89** was capable of racemizing the secondary amine at 60 °C, reaching 50% *ee* in 1.5 h and full racemization in 16 h. This would allow the application in enzymatic DKR reactions. Catalyst **89** was used for the racemization of a series of chiral amines; however, in the attempted racemization of primary amines, dimers were formed.

**Scheme 63:**
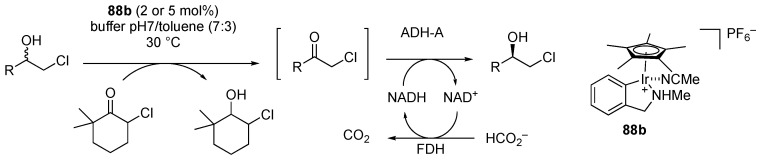
Deracemization of racemic chlorohydrines using combined iridacycle-catalyzed oxidation and enzyme-catalyzed reduction.

Kroutil developed a novel concept for a dynamic kinetic resolution [84]. The goal was to deracemize racemic chlorohydrins by oxidizing them in an Oppenauer oxidation catalysed by iridacycle **88b** to the chloroketones and, at the same time, use an alcohol dehydrogenase enzyme (ADH from *Rodococcus Ruber*) for the enantioselective reduction of the chloroketone to obtain the enantiopure chlorohydrin. The combination of an oxidation and a reduction in a single reaction is, of course, highly ambitious and, in practice, many hurdles had to be overcome. It was necessary for thermodynamic reasons to use a chloroketone as oxidant as, with simple aliphatic ketones, the equilibrium was too much on the side of the chlorohydrin. The initially chosen chloroacetone was an excellent oxidant for the iridacycle-catalyzed oxidation; however, unfortunately, it was also rapidly reduced by the enzyme. In the end, the sterically hindered 2-chloro-6,6-dimethylcyclohexanone, which is not a substrate for the enzyme, had to be used as oxidant. Next, the reductant for the enzyme had to be chosen such that it could not reduce the iridacycle catalyst. Luckily, this iridacycle could not be reduced by formic acid and, thus, the ADH was coupled with a formate dehydrogenase (FDH). Although the enzyme on its own was capable of reducing the substrates with 99% *ee*, this was unfortunately not achieved in the coupled system (Scheme 63). Three different substrates were subjected to the coupled reactions: 1-phenyl-2-chloro-ethanol (40% *ee*), 3-benzyloxy-1-chloracetone (6% *ee*), and 1-cyclohexyl-2-chloro-ethanol (29% *ee*). The lower *ee* was ascribed to the racemizing action of the iridacycle.

## 4. Conclusions

Ruthenacycles and iridacycles have been used for a large range of TH reactions. They form an interesting class that is characterized by the extremely easy synthesis of the catalyst and a surprisingly high stability. This allows for very high turnover numbers and turnover frequencies. The catalysts are made via cyclometalation of commercially available catalyst precursors in a single, usually high-yielding step. They have been applied for TH of ketones, imines, and nitrogen heterocycles, as well as for enantioselective variants thereof. They have also been used for the oxidation of alcohols, as Oppenauer oxidation catalyst, in combination with oxygen, and simply as dehydrogenation catalyst. In particular, this latter reaction has been used for the dehydrogenation of tetrahydro-quinolines and -isoquinolines, as well as indoles. Not surprisingly, they have also been used as racemization catalysts for alcohols and secondary and tertiary amines. This also allowed their use in dynamic kinetic resolutions in combination with lipases or haloalcohol dehalogenase enzymes. It is clear that many more applications of these catalysts can be expected in the near future, in particular of the chiral variants.

## Figures and Tables

**Table 1:** Rate comparison of iridacycles **54a**–**e** and **55a,b** in the TH of benzophenone.

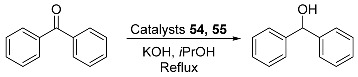				
Entry	Catalyst	Yield 2 h	Yield 4 h	Yield 24 h
1	**54a**	7	27	99
2	**55a**	4	28	99
3	**55b**	2	13	99
4	**54b**	77	99	n.d.
5	**54c**	30	96	99
6	**54d**	93	99	n.d.
7	**54e**	2	4	30

**Table 2:** Aerobic oxidative kinetic resolution of secondary alcohols.

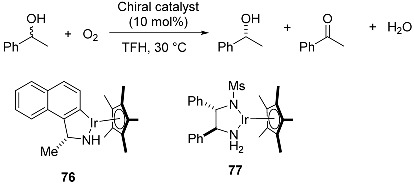				
Catalyst	Concentration of 1-phenylethanol	*t* (h)	Recovered Alcohol (%)	*ee* of Alcohol (%)
**76**	1.0 M	4	48	14
**76**	0.1 M	75	48	42
**77**	1.0 M	22	44	84
**77**	0.2 M	38	48	98
